# FOXA1 repression is associated with loss of BRCA1 and increased promoter methylation and chromatin silencing in breast cancer

**DOI:** 10.1038/onc.2014.421

**Published:** 2014-12-22

**Authors:** C Gong, K Fujino, L J Monteiro, A R Gomes, R Drost, H Davidson-Smith, S Takeda, U S Khoo, J Jonkers, D Sproul, E W-F Lam

**Affiliations:** 1 Department of Surgery and Cancer, Imperial College London, Hammersmith Hospital Campus, London, UK; 2 Department of Pathology, Li Ka Shing Faculty of Medicine, The University of Hong Kong, Hong Kong SAR, China; 3 Department of Obstetrics & Gynecology, Faculty of Medicine, Juntendo University, Bunkyoku, Tokyo, Japan; 4 Division of Molecular Pathology and Cancer Genomics Centre Netherlands, The Netherlands Cancer Institute, Amsterdam, The Netherlands; 5 MRC Human Genetics Unit, Institute of Genetics and Molecular Medicine, University of Edinburgh, Western General Hospital, Edinburgh, UK

## Abstract

FOXA1 expression correlates with the breast cancer luminal subtype and patient survival. RNA and protein analysis of a panel of breast cancer cell lines revealed that BRCA1 deficiency is associated with the downregulation of FOXA1 expression. Knockdown of BRCA1 resulted in the downregulation of FOXA1 expression and enhancement of *FOXA1* promoter methylation in MCF-7 breast cancer cells, whereas the reconstitution of BRCA1 in Brca1-deficent mouse mammary epithelial cells (MMECs) promoted *Foxa1* expression and methylation. These data suggest that BRCA1 suppresses *FOXA1* hypermethylation and silencing. Consistently, the treatment of MMECs with the DNA methylation inhibitor 5-aza-2′-deoxycitydine induced Foxa1 mRNA expression. Furthermore, treatment with GSK126, an inhibitor of EZH2 methyltransferase activity, induced FOXA1 expression in BRCA1-deficient but not in BRCA1-reconstituted MMECs. Likewise, the depletion of EZH2 by small interfering RNA enhanced FOXA1 mRNA expression. Chromatin immunoprecipitation (ChIP) analysis demonstrated that BRCA1, EZH2, DNA methyltransferases (DNMT)1/3a/3b and H3K27me3 are recruited to the endogenous *FOXA1* promoter, further supporting the hypothesis that these proteins interact to modulate *FOXA1* methylation and repression. Further co-immunoprecipitation and ChIP analysis showed that both BRCA1 and DNMT3b form complexes with EZH2 but not with each other, consistent with the notion that BRCA1 binds to EZH2 and negatively regulates its methyltransferase activity. We also found that EZH2 promotes and BRCA1 impairs the deposit of the gene silencing histone mark H3K27me3 on the *FOXA1* promoter. These associations were validated in a familial breast cancer patient cohort. Integrated analysis of the global gene methylation and expression profiles of a set of 33 familial breast tumours revealed that *FOXA1* promoter methylation is inversely correlated with the transcriptional expression of FOXA1 and that BRCA1 mutation breast cancer is significantly associated with FOXA1 methylation and downregulation of FOXA1 expression, providing physiological evidence to our findings that *FOXA1* expression is regulated by methylation and chromatin silencing and that BRCA1 maintains FOXA1 expression through suppressing FOXA1 gene methylation in breast cancer.

## Introduction

Breast cancer is the most common female malignancies worldwide, affecting one in nine women during their lifetime. Hereditary breast cancer constitutes 5–10% of all breast cancer cases. Together *BRCA1* and *BRCA2* mutations account for about 20–25% of all inherited breast cancers, and *BRCA1/2*-mutation carriers face a high risk of developing breast cancer. BRCA1 has diverse roles in breast cancer development and its multifaceted functions include DNA damage repair, cell cycle checkpoint control, protein ubiquitination and transcriptional control through chromatin modification and direct interaction with RNA polymerase II holoenzyme complex.^1^ The vast majority of BRCA1-deficient tumours exhibit basal-like breast cancer phenotypes, which include triple-negative receptor status (ER-, PR- and HER2-negative), strong expression of basal cytokeratins, high p53 mutation rates and poor prognosis.^2^ Consistently, the loss of BRCA1 hinders differentiation into ER-positive luminal cells, leading to increased stem/progenitor cell-like self-renewal properties.^3, 4^ Nevertheless, the exact mechanism by which BRCA1 regulates the differentiation of luminal breast cancer cells is still poorly understood. It has been suggested that BRCA1 might regulate luminal differentiation through the transcriptional activation of ER.^5^ BRCA1 might also regulate mammary cell fate through transcriptional activation of Notch signalling pathway.^2^ In addition, BRCA1 could also interact with GATA3 at its C-terminal region to repress triple-negative and basal-like breast cancer (BLBCs) associated genes, such as *FOXC1*.^6^

FOXA1 is a ‘pioneer' forkhead transcription factor that can directly bind condensed chromatin, displace repressive linker histones and recruit other transcription factors to promote transcription.^7, 8^ It is required by ERα as a cofactor for chromatin binding^9^ and has been reported to regulate nearly 50% of all oestrogen receptor (ER)-target genes.^10^ Thus, FOXA1 and ERα constitute a major proliferative and survival axis for breast cancers, specifically of the luminal A subtype. Crucially, FOXA1 is a favourable prognostic marker^10, 11, 12^ and can directly repress the expression of a subset of basal specific genes. Accordingly, the silencing of FOXA1 causes a partial transcriptome shift from luminal to basal gene expression signatures.^13^ FOXA1 may also prevent metastatic progression of luminal subtype breast cancers by controlling differentiation through enhancing the expression of the negative cell cycle regulator p27^Kip1^ and the cell adhesion molecule E-cadherin.^14^

EZH2 (enhancer of zeste homologue 2) is a subunit of the polycomb-repressive complex 2 (PRC2) and its overexpression is also associated with the presence of metastasis and poor survival in breast cancer patients. EZH2 mediates the trimethylation of histone 3 lysine 27 (H3K27me3), which serves as an epigenetic code for subsequent recruitment of PRC1, DNA methyltransferases (DNMTs) and histone deacetylases, resulting in chromatin condensation and transcription suppression.^15^ In addition, EZH2 can also directly interact with DNMTs and regulate the methylation of EZH2-target genes,^16^ although these interactions might be cell-type specific.^17^ Recently, EZH2 has also been functionally linked with BRCA1 in breast cancer. It has been shown that the growth of BRCA1-deficient mouse mammary tumours is dependant on EZH2 expression.^18^ Conversely, EZH2 inhibition in breast cancer cell lines can cause BRCA1 nuclear exportation and inactivation.^19^ Crucially, BRCA1 interacts directly with EZH2 in both mouse embryonic stem cells and human breast cancer cells, functioning as a negative modulator of EZH2 activity.^20^

In this study, we hypothesized that BRCA1 regulates FOXA1 expression and confirmed it by the reconstitution or knockdown of BRCA1. We found that BRCA1 deficiency correlates with the repression of FOXA1 expression in mammary epithelial cancer cell lines and that BRCA1 mutation is significantly associated with *FOXA1* promoter methylation and silencing in human breast tumours. We also went on to explore the potential molecular mechanism involved and identified *FOXA1* as an EZH2-regulated gene in breast cancer cells.

## Results

### Correlation between BRCA1 and FOXA1 expression in breast cancer cell lines

**Figure 1:**
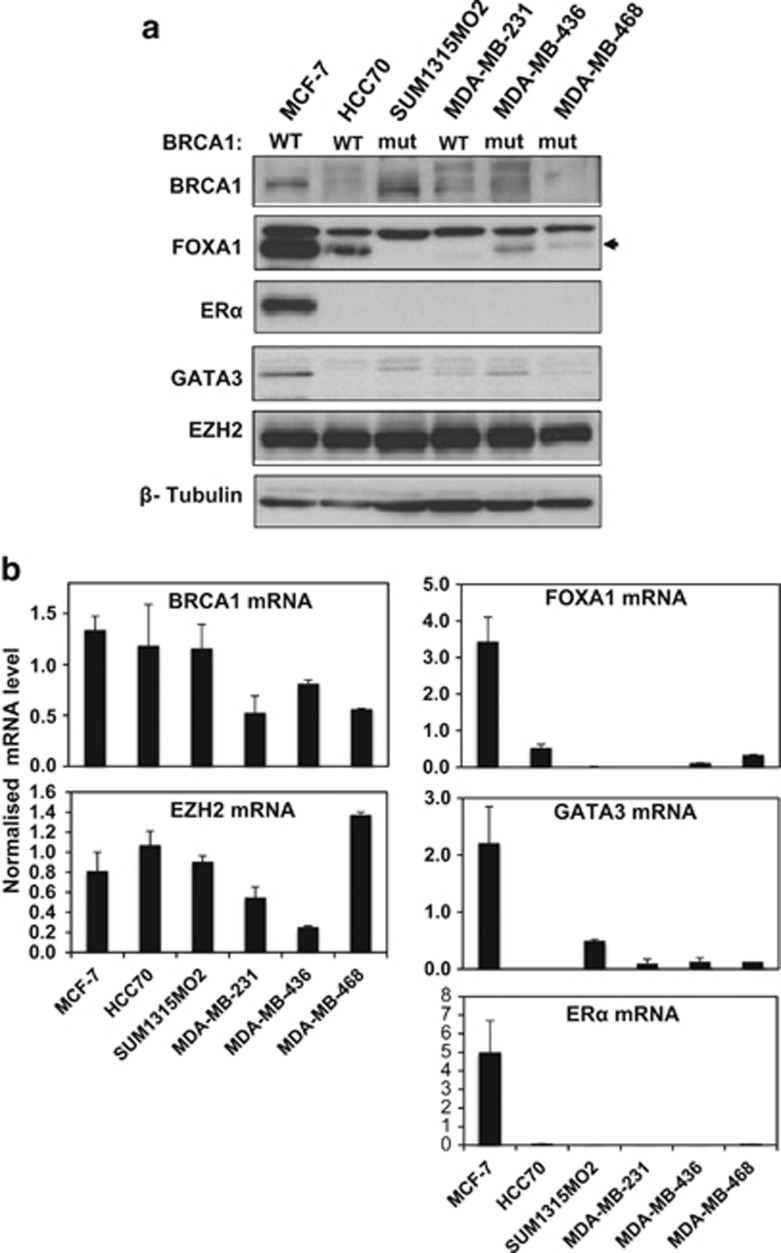
BRCA1 correlates with FOXA1 expression in breast cancer cell panel. (**a**) Western blot and (**b**) quantitative reverse transcription-PCR (qRT–PCR) analysis was performed on a panel of six different breast cancer cell lines including the luminal-type cell line MCF-7, which expresses wild-type BRCA1, basal-type cell lines HCC70, MDA-MB-231, MDA-MB-436, SUM1315MO2 and MDA-MB-468 expressing either low or mutated BRCA1. The experiments were repeated three times independently and qRT–PCR results were normalized against L19 mRNA levels and the results presented as bars representing mean±s.d.

The majority of BRCA1 mutation breast cancers are of the basal subtype. The molecular phenotype of basal breast cancers has been shown to be repressed by FOXA1 expression, which is associated with the luminal phenotype.^13^ These findings raise the possibility that BRCA1 and FOXA1 interact at the molecular level to modulate the development of basal and luminal breast cancer subtypes. To investigate the relationship between BRCA1 and FOXA1, western blot analysis was performed on a panel of six breast cancer cell lines, which include the luminal-type MCF-7 cells with wild-type BRCA1 and the basal-type lines HCC70, MDA-MB-231, MDA-MB-436, SUM1315MO2 and MDA-MB-468 expressing either low or mutated BRCA1^21^ (Figure 1a). Despite the great heterogeneity among the cell lines, there was a good correlation between the expression of wild-type BRCA1 and FOXA1. Similar correlations between BRCA1 and FOXA1 expression were observed in a panel of breast cancer cell lines with different levels of wild-type BRCA1 (Supplementary Figure S1). In the basal-type cell lines, FOXA1 expression was much lower when compared with that of MCF-7, which supports the notion that FOXA1 is a marker for luminal subtype breast cancer.^13^ The expression of FOXA1 in MCF-7 cells was also significantly higher compared with the basal-type cell lines at the mRNA level (Figure 1b). Consistent with this, other luminal markers, ERα and GATA3, were also only expressed in considerable levels in MCF-7 cells. We also studied the expression of the Polycomb protein EZH2, which has previously been demonstrated to interact with BRCA1^20^ and found it ubiquitously expressed in all six cell lines. Notably, high BRCA1 mRNA levels, which encode for non-functional BRCA1, were also detected in some of the BRCA1 mutation cell lines.

### BRCA1 regulates FOXA1 expression in human and mouse mammary epithelial cells

**Figure 2:**
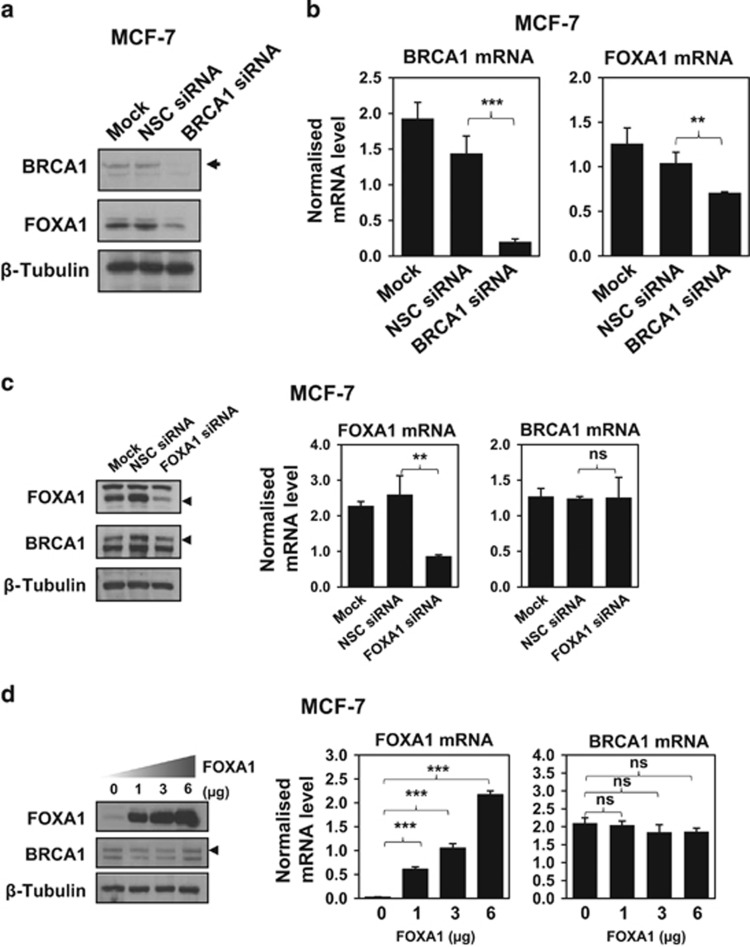
Knockdown of BRCA1 results in a significant decrease in FOXA1 expression and not vice versa in MCF-7 cells. (**a**) Western blot and (**b**) quantitative reverse transcription-PCR (qRT–PCR) analysis was performed MCF-7 cells transfected with BRCA1-specific siRNA pool for 48 h. FOXA1 expression was observed to be downregulated both at the protein and mRNA levels. For qRT–PCR analysis, the experiments were repeated three times independently and the results were normalized against L19 mRNA levels and expressed as mean±s.d. **P⩽0.01, ***P⩽0.001 and NS, no significance by Student's *t*-test. (**c**) MCF-7 cells were either non-transfected (Mock) or transfected with NSC siRNA or with FOXA1 siRNA-specific pool for 48 h. The protein expression levels of FOXA1, BRCA1 and β-Tubulin were assessed by western blot analysis (arrows indicate the specific protein band). Levels of FOXA1, BRCA1 and FOXM1 transcripts were analyzed by qRT–PCR normalized with L19 mRNA levels. (**d**) MCF-7 cells were transfected with 0, 1, 3 or 6 μg of pcDNA3-FOXA1 or pcDNA3-empty vector for 48 h. Total protein was extracted from whole-cell lysates were extracted from these cells and analyzed by western blotting with the indicated antibodies (arrow indicates the specific protein band). FOXA1 and BRCA1 mRNA levels were also analyzed by qRT–PCR, with results normalized with L19 mRNA levels. All qRT–PCR results presented as bars representing mean±s.d. of three independent experiments in triplicates. **P⩽0.01, ***P⩽0.001 and NS indicates no significance by Student's *t*-test.

The good correlation between BRCA1 and FOXA1 expression in the panel of breast cancer cell lines led us to explore further the possibility that BRCA1 regulates FOXA1 expression. To this end, BRCA1 was silenced in MCF-7 cells using BRCA1-specific small interfering RNA (siRNA) pool. BRCA1 depletion resulted in a significant reduction in FOXA1 expression at both protein (Figure 2a) and mRNA levels in MCF-7 cells (Figure 2b) when compared with the non-silencing control (NSC), supporting the notion that BRCA1 regulates FOXA1 expression. Conversely, the knockdown or overexpression of FOXA1 did not affect BRCA1 protein and mRNA levels (Figures 2c and d). Taken together, these results indicate that BRCA1 regulates FOXA1 expression and not vice versa, and that BRCA1 regulates FOXA1 expression, at least in part, at the transcriptional level.

**Figure 3:**
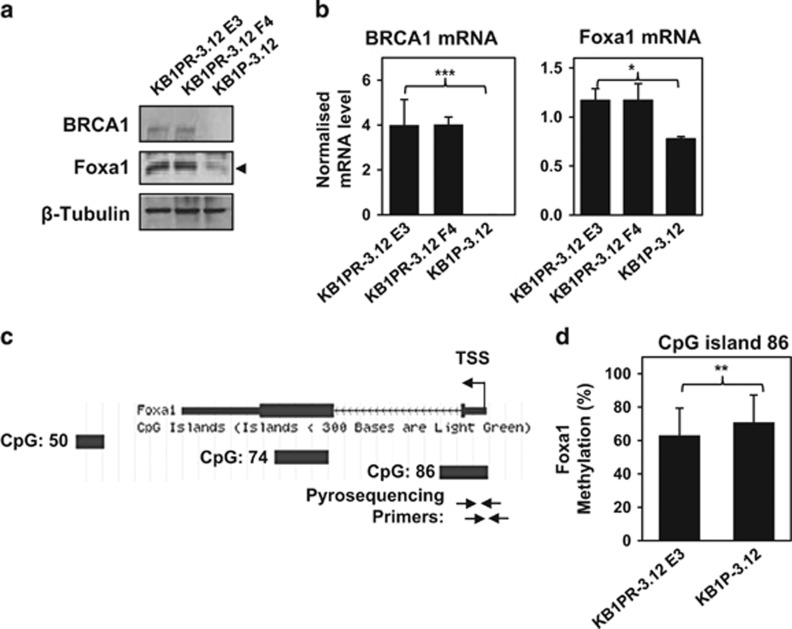
FOXA1 expression is lower and its promoter more hypermethylated in the BRCA1-deficient murine mammary epithelial cell line. (**a**) KB1PR-3.12 E3, KB1PR-3.12 F4 and KB1P-3.12 murine mammary epithelial cell lines were collected and analyzed by western blotting and (**b**) quantitative reverse transcription-PCR (qRT–PCR) to determine the expression levels of BRCA1 and FOXA1. qRT–PCR results are presented as the mean±s.d. of three independent experiments in triplicates. (**c**) Schematic representation, as presented on UCSC genome web browser, of the locations of CpG islands in mouse *Foxa1* gene and the positions of primers used for pyrosequencing analysis. (**d**) DNA extracts from KB1PR-3.12 E3 and KB1P-3.12 cell lines were bisulphite converted and methylation status of *Foxa1* promoter region were analyzed by pyrosequencing. Average *Foxa1* methylation values of the two analyzed regions within the CpG island 86, located in the Foxa1 promoter region, are shown for both cell lines. Results are expressed as the mean±s.d. from two independent experiments in triplicates. *P⩽0.05 **P⩽0.01, ***P⩽0.001 by Student's *t*-test. TSS, Transcription start site.

To confirm further that the loss of FOXA1 expression is a result of BRCA1 depletion in mammary epithelial cells, we next investigated the effect of re-expressing BRCA1 on FOXA1 expression in Brca1-deficient mouse mammary epithelial cells (MMECs). Analysis of the expression levels of Foxa1 in Brca1-deficient KB1P-3.12 (K14cre;Brca1F/F;p53F/F, clone 12) and BRCA1-reconstituted KB1PR-3.12 E3 and KB1PR-3.12 F4 (K14cre;Brca1F/F;p53F/F BRCA1-reconstituted clones E3 and F4) MMECs,^18^ revealed that Foxa1 expression was significantly higher at both the mRNA and protein levels in the BRCA1-reconstituted cell lines compared with the parental Brca1-deficient, KB1P-3 12 cells (Figures 3a and b), further confirming that BRCA1 has a role in modulating FOXA1 expression.

### FOXA1 promoter is hypermethylated in the BRCA1-deficient MMECs compared with the BRCA1-resconstituted cells

DNA methylation is a well-defined epigenetic mechanism for the repression of gene expression^22^ and BRCA1 mutation is associated with specific global DNA methylation profiles in breast cancer.^23^ These findings suggested that BRCA1 deficiency may lead to DNA methylation and therefore the silencing of *FOXA1*. To test this conjecture, we studied the extent of DNA methylation in the CpG islands located within the *Foxa1* gene promoter in the Brca1-deficient mouse epithelial cells (Figure 3c). *In silico* analysis using the UCSC Genome browser website (www.genome.ucsc.edu) predicted three regions of CpG sites (CpG 50, CpG 74 and CpG 86) within the mouse *Foxa1* gene. Bisulphite pyrosequencing revealed that the Brca1-deficient KB1P-3.12 cells displayed significantly higher levels of DNA methylation at the CpG island 86 that locates nearby the mouse *Foxa1* promoter region when compared with the Brca1-knockout cells reconstituted with BRCA1 (Figure 3d). This suggests that *Foxa1* promoter methylation might contribute to the lower Foxa1 expression observed in the KB1P-3.12 cells (Figures 3a and b).

**Figure 4:**
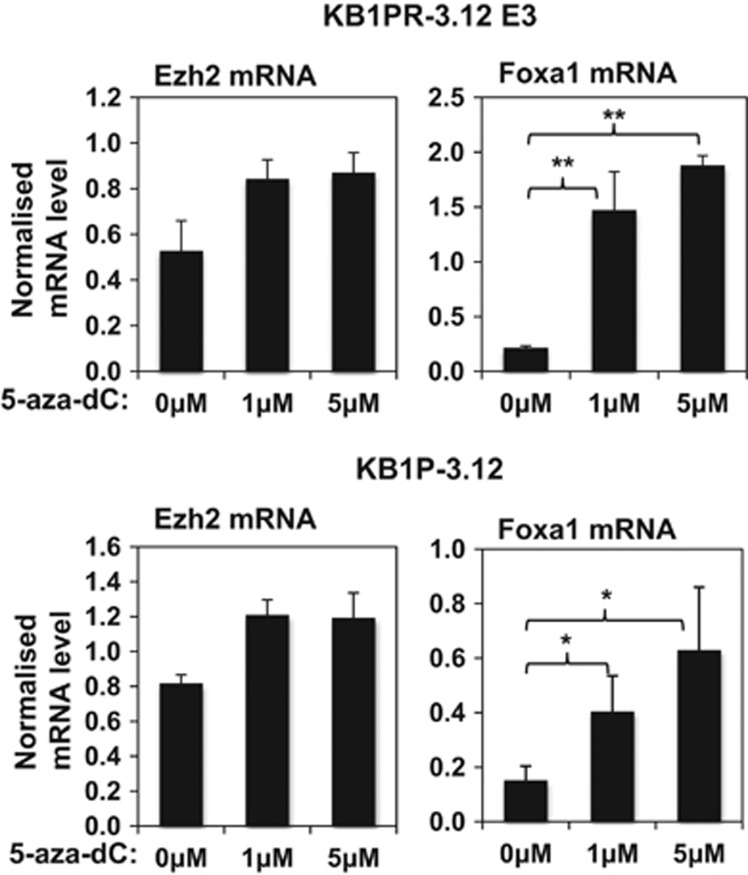
5'-Aza-dC treatment induces *Foxa1* mRNA expression in KB1PR-3.12 E3 and KB1P-3.12. The two cell lines were treated with 0, 1  and 5 μM of 5'-aza-dC for 72 h with culture medium changed every day. Total RNA was extracted and expression of Ezh2 and Foxa1 mRNA was analyzed by quantitative reverse transcription-PCR (qRT–PCR). The experiments were repeated three times independently and qRT–PCR results were normalized against L19 mRNA levels and the results expressed as mean±s.d. *P⩽0.05 and **P⩽0.001 by Student's *t*-test.

Consistently, Foxa1 transcript levels were significantly induced when treating the KB1P-3.12 and KB1PR-3.12 E3 cells with 5-aza-2'-deoxycytidine (5-aza-dC), a drug that inhibits the activity of DNMT and suppresses DNA methylation^24^ (Figure 4), supporting the notion that *Foxa1* promoter is methylated in Brca1-deficient cells. However, a corresponding induction of FOXA1 at the protein level was not observed after 5'-aza-dC treatment implicating other mechanisms exist to regulate FOXA1 post-transcriptionally in these cells (Supplementary Figure S2).

### Knockdown of BRCA1 in MFC-7 induces FOXA1 promoter methylation

**Figure 5:**
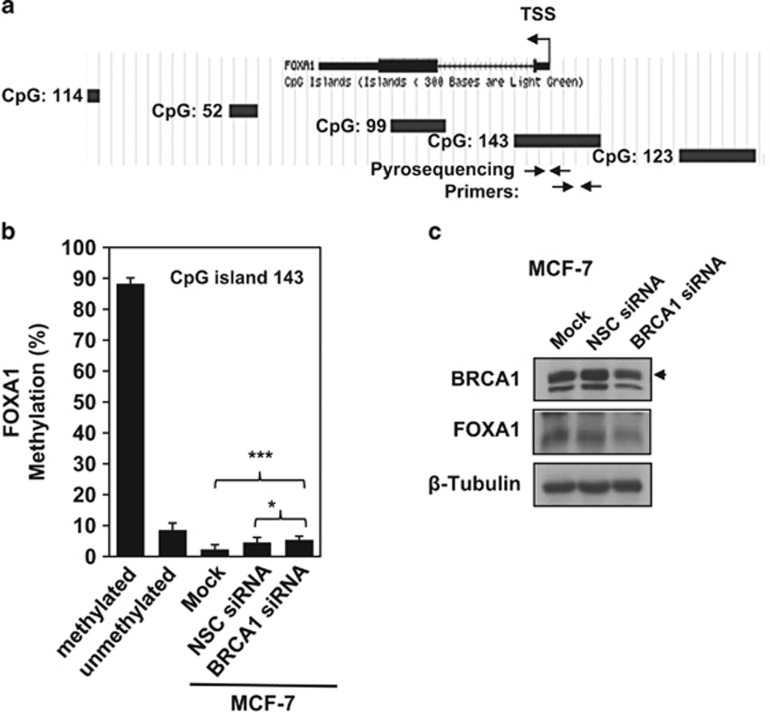
Knockdown BRCA1 in MFC-7 cells induces FOXA1 promoter methylation. (**a**) Schematic representation, as presented on UCSC genome web browser, of the location of CpG islands in the human *FOXA1* gene and the position of primers used for pyrosequencing analysis. (**b**) MCF-7 cells were either non-transfected (Mock) or transfected with NSC siRNA or with FOXA1 siRNA-specific pool. Forty-eight hours post transfection, DNA was extracted, bisulphite converted and analyzed by pyrosequencing. Average FOXA1 methylation values of the two analyzed regions within the CpG island 143, located in the FOXA1 promoter region, are shown. (**c**) Western blot analysis was performed to determine protein expression of BRCA1, FOXA1 and β-Tubulin (arrows indicate specific protein band) in MCF-7 cells. Results are presented as the mean±s.d. from two independent experiments in triplicates. *P<0.05, ***P⩽0.001 by Student's *t*-test. TSS, Transcription start site.

In the human *FOXA1* gene, five CpG islands (CpG 110, CpG 52, CpG 99, CpG 143 and CpG 123) were identified. We focused on CpG island 143 as it corresponds to CpG island 86 located at the promoter region of the mouse *Foxa1* gene (Figure 5a). MCF-7 was transiently transfected with either NSC or BRCA1 siRNA, and FOXA1 methylation levels analyzed. Methylated and unmethylated DNAs were also used in the pyrosequencing experiment. In the MCF-7 cells, the *FOXA1* promoter was hypomethylated. Knockdown of BRCA1 led to significantly higher levels of *FOXA1* methylation compared with the mock (non-transfected) and the NSC siRNA-transfected controls (Figure 5b). Western blot analysis confirmed the knockdown of BRCA1 in MCF-7 cell lines and FOXA1 expression was reduced by BRCA1 knockdown (Figure 5c). Collectively, these data suggest that BRCA1 positively regulates FOXA1 expression, in part, through suppressing its promoter methylation. However, the relatively low levels of DNA methylation before and after 5'-aza-dC treatment suggested that the *FOXA1* promoter in the MCF-7 cell line is undermethylated.

### 5′-aza-dC treatment induces FOXA1 expression in basal-type cell lines

**Figure 6:**
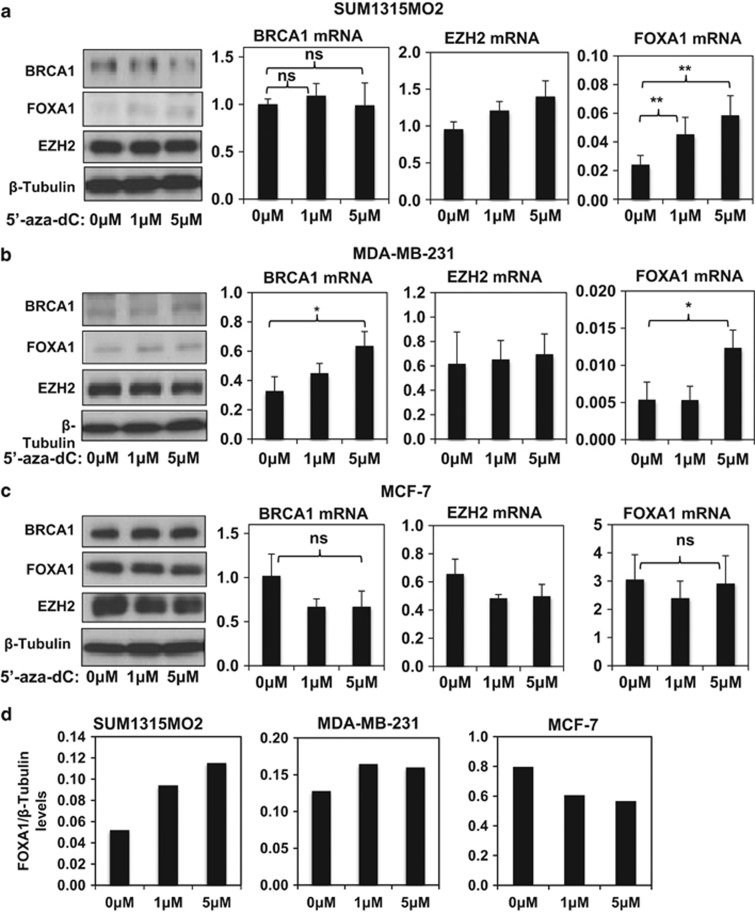
5'-Aza-dC treatment induces FOXA1 expression in basal-type breast cancer cell lines. The basal-type cell lines SUM1316MO2 and MDA-MB231 and the luminal-type cell line MCF-7 were treated with increasing amount of 5′-aza-dC (0, 1 and 5 μM) for 72 h and BRCA1, FOXA1 and EZH2 expression levels were examined by western blot and quantitative reverse transcription-PCR (qRT–PCR) analysis. Treatment of 5′-aza-dC induced expression of FOXA1 in (**a**) SUM1316MO2 and (**b**) MDA-MB231 but not in (**c**) MCF-7 cells. The experiments were repeated three times independently and qRT–PCR results were normalized against L19 mRNA levels and the results presented as average±s.d. (**d**) Quantitative analysis of FOXA1 protein expression in SUM1316MO2, MDA-MB231 and MCF-7 cells following treatment with 5'-aza-dC (0, 1 and 5 μM) was performed using Image J Software (Image Processing and Analysis in Java). The values were normalized against those of β-Tubulin. *P⩽0.05, **P⩽0.01 and NS indicates no significance by Student's t-test.

To investigate further the relevance of *FOXA1* methylation in repressing FOXA1 expression in basal breast cancer, the basal-type cell lines SUM1316MO2 and MDA-MB-231 and the luminal-type cell line MCF-7 were treated with different amounts of 5′-aza-dC (0, 1 and 5 μM) for 72 h and BRCA1, FOXA1 and EZH2 expression levels examined by both western blot and quantitative reverse transcription-PCR. In SUM1315MO2, FOXA1 mRNA expression was significantly induced after treatment with 1 and 5 μM of 5′-aza-dC treatment (both *P*<0.05, Student's *t*-test) (Figure 6a). In agreement, the FOXA1 expression was also induced after being treated with 5 μM of 5′-aza-dC in MDA-MB-231 cells (Figure 6b). Notably, the expression of BRCA1 at the mRNA level also increased significantly (*P*<0.05, Student's *t*-test) after 5'-aza-dC treatment, suggesting that DNA methylation contributes to the low BRCA1 expression in this cell line. In fact, promoter methylation has been reported to be a regulatory mechanism for BRCA1 expression and is associated with poor prognosis in breast cancer.^25, 26^ On the contrary, treatment of the luminal MCF-7 with 5'-aza-dC did not alter the expression levels of FOXA1 (Figure 6c) indicating that, in the presence of wild-type BRCA1, FOXA1 expression is not repressed by DNA methylation. Collectively, these results suggest that DNA methylation has a part in low *FOXA1* expression in basal subtype breast cancers where BRCA1 is either mutated or depleted (Figure 6d), highlighting a role of BRCA1 in promoting FOXA1 expression through suppressing *FOXA1* methylation in luminal breast cancers.

### Inhibition or depletion of EZH2 induces FOXA1 expression in BRCA1-deficient mammary epithelial cells

**Figure 7:**
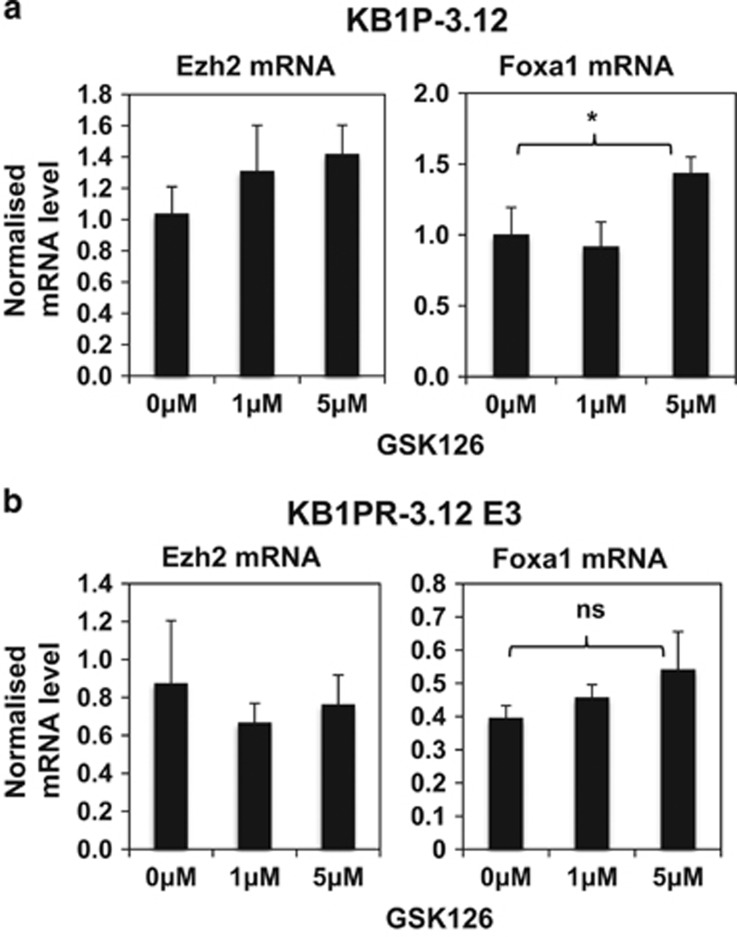
EZH2 inhibition by GSK126 treatment induces *Foxa1* expression in murine BRCA1-deficient murine mammary epithelial cell line. The BRCA1-deficient murine mammary epithelial cell line KB1P-3.12 and the corresponding BRCA1-reconstituted murine cell line KB1PR-3.12 E3 were treated with 0, 1 or 5 μM of GSK126 for 72 h. Total RNA was collected and expression of *Ezh2* and *Foxa1* examined by quantitative reverse transcription-PCR (qRT–PCR). (**a**) *Foxa1* expression at the mRNA level was significantly induced after treatment with 5 μM of GSK126 in KB1P-3.12 cells. (**b**) However, *Foxa1* mRNA expression did not show significant changes in the BRCA1-reconstituted murine cell line KB1PR-3.12 E3 after GSK126 treatment. The experiments were repeated three times independently and qRT–PCR results normalized against L19 mRNA levels and the results expressed as mean±s.d. *P⩽0.05 and NS indicates no significance by Student's *t*-test.

**Figure 8:**
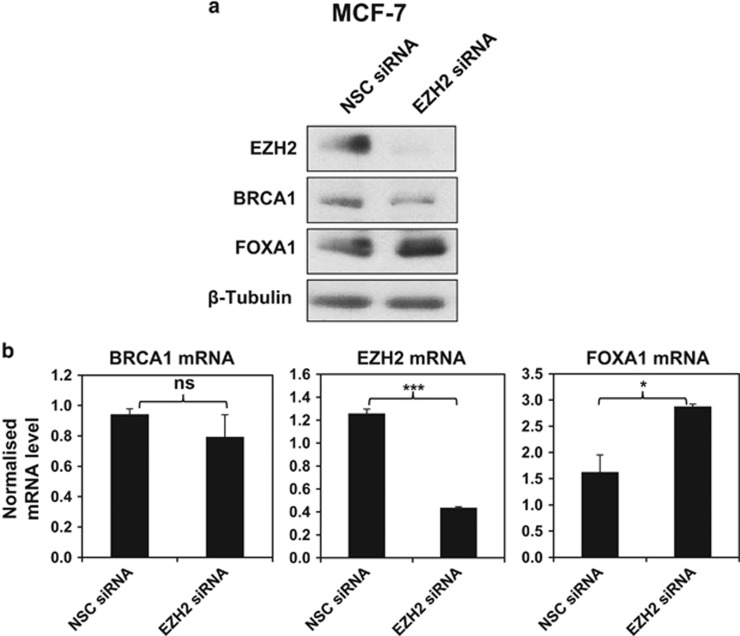
Knockdown of EZH2 in MCF-7 cells induces FOXA1 expression. EZH2 was transiently knocked down using specific siRNA pool in MCF-7 for 48 h. Protein and total RNA were extracted and expression levels of EZH2, BRCA1 and FOXA1 analyzed by (**a**) western blot and (**b**) quantitative reverse transcription-PCR (qRT–PCR) analysis. Knockdown of EZH2 significantly induced FOXA1 expression at both protein and mRNA levels. The experiments were repeated three times independently and qRT–PCR results were normalized against L19 mRNA levels and the results expressed as mean±s.d. *P⩽0.05, ***P⩽0.001 and NS indicates no significance by Student's t-test.

We next explored the mechanism by which BRCA1 regulates *FOXA1* methylation and the cofactors involved. A recent study showed that BRCA1 interacts with the PRC2 protein EZH2 to negatively regulate gene expression through promoting histone H3 lysine 27 trimethylation (H3K27me3) in mouse embryonic stem and human breast cancer cells.^20^ These findings raised the possibility that BRCA1 modulates *FOXA1* methylation and expression through recruiting EZH2. To test this idea, we treated the Brca1-deficient KB1P-3.12 murine mammary epithelial cells and the corresponding BRCA1-reconstituted KB1PR-3.12 E3 cells for 72 h with GSK126, a highly selective, *S*-adenosyl-methionine-competitive inhibitor of EZH2 methyltransferase activity^27^ and studied its effect on Foxa1 expression. The results showed that Foxa1 transcript levels were significantly induced by treatment with 5 μM of GSK126 in the BRCA1-deficient KB1P-3.12 cells (*P*<0.05, Student's *t*-test, Figure 7a) but remained largely constant in the BRCA1-reconstituted KB1PR-3.12 E3 cells (Figure 7b). This result suggests that EZH2 is involved in the repression of FOXA1 expression in the BRCA1-deficient cells but not in the BRCA1-competent cells. However, there were no changes in FOXA1 protein levels after treatment with GSK126, indicating that FOXA1 is also regulated at the post-transcriptional level, consistent with the previous 5'aza-dC treatment results with these murine cell lines (Supplementary Figure S3). To further confirm this finding, we used siRNA to deplete EZH2 in the human breast cancer MCF-7 cells. The knockdown efficiency of EZH2 after 48 h was confirmed by western blot and quantitative reverse transcription-PCR analysis. It was found that after EZH2 depletion FOXA1 expression was significantly induced both at protein and mRNA levels when compared with the NSC (Figures 8a and b). These data corroborate with the hypothesis that EZH2 negatively regulates *FOXA1* transcription in BRCA1-deficient breast cancer cells.

### EZH2 directly interacts with DNMT3b and binds to FOXA1 promoter

**Figure 9:**
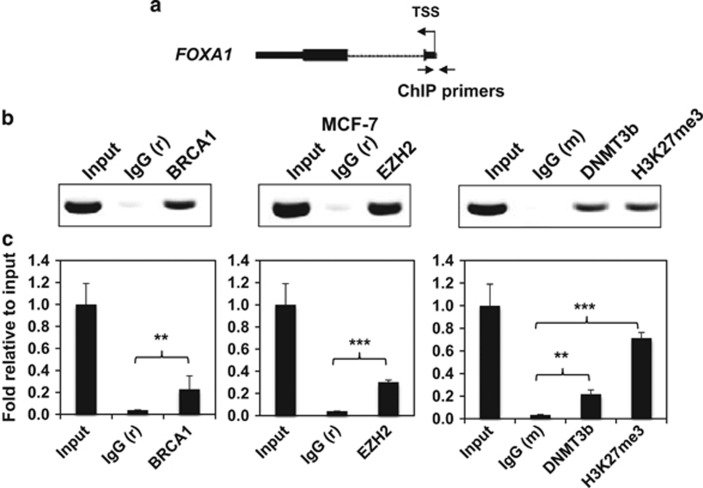
EZH2 is recruited to the *FOXA1* promoter and complexes with BRCA1 and DNMT3b. MCF-7 cells were analyzed for of EZH2, DNMT3b and H3k27me3 to the endogenous *FOXA1* promoter by ChIP assays. The PCR-amplified DNA was analyzed by agarose gel electrophoresis. A representative inverted image is presented. (**a**) Schematic representation, as presented on UCSC genome web browser, of the human FOXA1 gene and the position of primers used for ChIP analysis. (**b**) The precipitated chromatin DNA was quantified by quantitative reverse transcription-PCR (qRT–PCR). (**c**) The results were normalized to the amount of Input and compared with the IgG negative controls. The experiments were repeated three times independently and the qRT–PCR results presented as mean±s.d. **P⩽0.01 and ***P⩽0.001 by Students' *t*-test. IgG (r), rabbit IgG negative control. IgG (m), mouse IgG negative control.

Hitherto, our data indicated that BRCA1 inhibits EZH2 activity to suppress the methylation and silencing of *FOXA1*. Previously, EZH2 has been shown to cause Histone 3 lysine 27 trimethylation (H3K27me3) and DNA methylation at target genes by recruiting DNMTs.^16^ Collectively, these findings evoked the idea of EZH2 inducing H3K27 trimethylation and recruiting DNMTs to promote heterochromatinization and DNA methylation respectively at the *FOXA1* promoter in BRCA1-deficient cells. To test this possibility, we next investigated by ChIP analysis the *in vivo* occupancy of the human *FOXA1* promoter region by BRCA1, EZH2, DNMT3b and H3K27me3 in the BRCA1-positive MCF-7 cells, where the *FOXA1* promoter is hypomethylated. Incidentally, the inspection of this human *FOXA1* promoter region also identified a putative EZH2-binding motif.^28^ Primers for ChIP were designed to amplify across the predicted EZH2-binding region of *FOXA1* promoter (Figure 9a). The ChIP results showed that BRCA1, EZH2, DNMT3b and H3K27me3 can all bind to the *FOXA1* promoter in MCF-7 cells (Figure 9b). The protein recruitment to the endogenous *FOXA1* promoter was also examined by quantitative reverse transcription-PCR following ChIP (Figure 9c). The results again demonstrated that BRCA1, EZH2, DNMT3b and H3K27me3 are specifically associated with the endogenous *FOXA1* promoter region further supporting our hypothesis that these proteins interact to modulate *FOXA1* methylation and silencing. To further confirm this, the methylation status of the *FOXA1* promoter was examined in the BRCA1 wild-type and -deficient breast epithelial cell lines by PCR analysis of bisulfite-converted DNA (Supplementary Figure S4). Surprisingly, the *FOXA1* promoter region was hypomethylated in both BRCA1 wild-type and deficient human cell lines. Gene promoter hypomethylation has previously been shown to be due to the prolonged passaging of cell lines and the immortalization process^29^ and could explain for the inefficient derepression of *FOXA1* expression in BRCA1-deficient human breast cancer cell lines after 5'-aza-dC treatment.

### BRCA1 represses and EZH2 enhances the deposit of the H3K27me3 gene silencing histone marker

**Figure 10:**
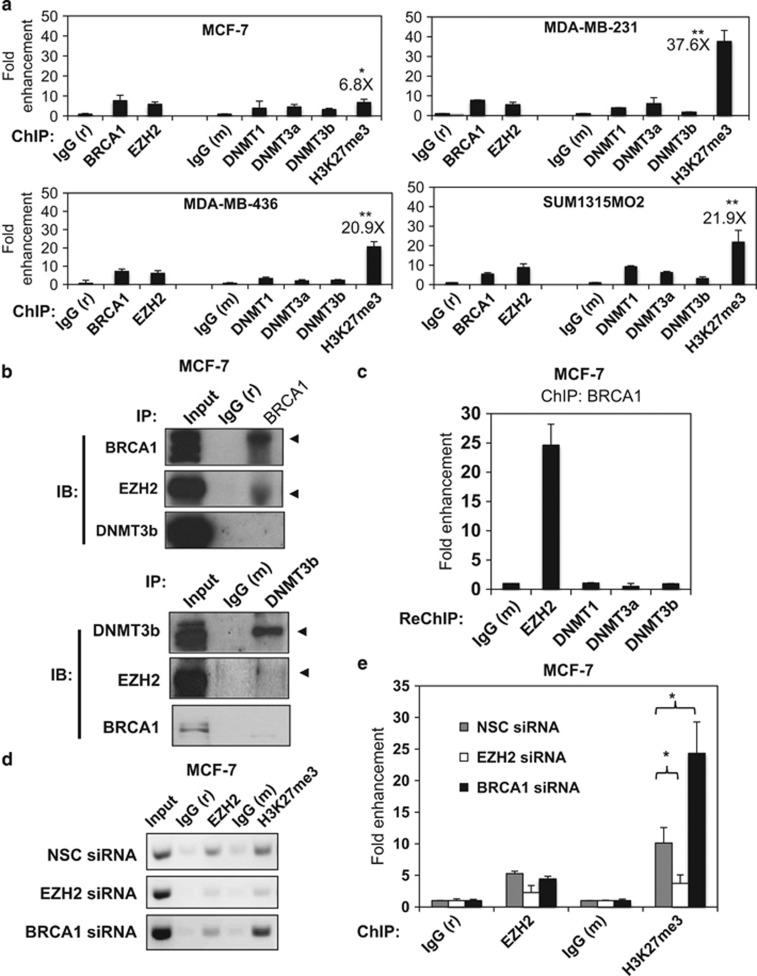
EZH2 but not BRCA1 recruits DNMT3b and H3K27me3 to the *FOXA1* promoter. (**a**) quantitative reverse transcription-PCR (qRT–PCR) analysis of ChIP showing the recruitment of BRCA1, EZH2, DNMT1, DNMT3a, DNMT3b and H3K27me3 to the endogenous *FOXA1* promoter in MCF-7, MDA-MB-231, MDA-MB-436 and SUM1315MO2 cells. The ChIP results showed that BRCA1, EZH2, DNMT1, DNMT3a and DNMT3b were all recruited to the FOXA1 promoter albeit at low levels (all with *P<0.05 compared with IgG controls). The fold of enrichment over IgG (mouse) is shown for ChIP with antibodies against H3K27me3. The experiments were repeated three times independently and the qRT–PCR results presented as mean±s.d. **P⩽0.01 by Student's *t*-test. (**b**) BRCA1 and DNMT3b were immunoprecipitated using specific antibodies and the precipitated proteins analyzed by western blot analysis. The specific bands were indicated by arrows. Co-immunoprecipitation (Co-IP) showed that, in MCF-7 cells, EZH2 associated with BRCA1 and DNMT3b but not in the same complexes. (**c**) ChIP–reChIP assays were performed with BRCA1 and EZH2, DNMT1, DNMT3a or DNMT3a antibodies in MCF-7. (**d**) ChIP analysis was performed on MCF-7 cells after BRCA1 or EZH2 silencing by siRNA and recruitment of BRCA1, EZH2, and H3K27me3 to the endogenous *FOXA1* promoter MCF-7 cells were transiently transfected with smart pool siRNA against BRCA1, EZH2 or control siRNA pool. Twenty-four hours later, the transfected MCF-7 cells were analyzed for EZH2 and H3K27me3 recruitment to the *FOXA1* promoter by ChIP assays. The precipitated chromatin DNA was analyzed by conventional PCR (**e**) The precipitated chromatin DNA was quantified by qRT–PCR. The results were normalized to the amount of Input and compared with the IgG negative controls. The experiments were repeated three times independently and the qRT–PCR results presented as mean±s.d. *P⩽0.05, **P⩽0.01 and ***P⩽0.001 by Student's *t*-test. IgG (r), rabbit IgG negative control. IgG (m), mouse IgG negative control.

To investigate further the mechanism involved in FOXA1 repression in BRCA1-deficient cells, we studied the recruitment of BRCA1, EZH2, DNMT1/3a/3b and H3K27me3 to the endogenous *FOXA1* promoter in MCF-7, MDA-MB-231, SUM131MO2 and MDA-MB-436 cells by ChIP analysis. Consistent with previous data, the ChIP results showed that BRCA1, EZH2, DNMT1, DNMT3a and DNMT3b were all recruited to the *FOXA1* promoter albeit at low levels (all with *P*<0.05 compared with respective IgG controls; Figure 10a). Interestingly, high levels of the repressive H3K27me3 histone marks were detected at the *FOXA1* promoter in the BRCA1-deficient MDA-MB-231, SUM131MO2 and MDA-MB-436 compared with the wild-type BRCA1-expressing MCF-7 cells, suggesting that gene silencing via heterochromatinization could be the predominant mechanism by which *FOXA1* expression is repressed in the hypomethylated BRCA1-deficient cells. To gain further information on the spatial relationships between these proteins in the regulation of *FOXA1* repression, we first studied the interactions of BRCA1, EZH2 and DNMT3b in MCF-7 cells by co-immunoprecipitation. The result showed that both BRCA1 and DNMT3b co-precipitated with EZH2 but not with each other. These co-immunoprecipitation data led us to propose that the BRCA1-containing EZH2 complexes do not contain DNMTs, such as DNMT3b, and are therefore inactive in DNMT activity. To validate this, we performed sequential ChIP (ChIP–reChIP) experiments in MCF-7 cells and found that BRCA1 co-occupied the *FOXA1* promoter region with EZH2 but not DNMT1/3a/3b (Figure 10b). ChIP–reChIP experiments also showed that the levels of DNMT3b recruited with EZH2 to the *FOXA1* promoter were significantly higher in MDA-MB-231 compared with MCF-7 cells (Supplementary Figure S5). We next examined the role of BRCA1 and EZH2 in the deposit of the histone marker H3K27me3 in MCF-7 cells by ChIP analysis after BRCA1 or EZH2 silencing. Interestingly, both the conventional ChIP (Figure 10c) and qPCR-ChIP (Figure 10d) analysis showed that BRCA1 depletion enhanced and EZH2 knockdown reduced H3K27me3 levels on the *FOXA1* promoter. This is in agreement with a previous study showing that BRCA1 binds directly to EZH2 and inhibits PRC2 activity,^20^ which regulated histone methylation and the DNA methylation of target genes.^16^ Consistent with this idea, the expression of BRCA1 could significantly upregulate FOXA1 mRNA expression in basal-like breast cancer cells, including MDA-MB-231 and MDA-MB-436, but not in the luminal cells, such as MCF-7 (Supplementary Figure S6).

### FOXA1 is hypermethylated and downregulated in BRCA1-mutated tumours

**Figure 11:**
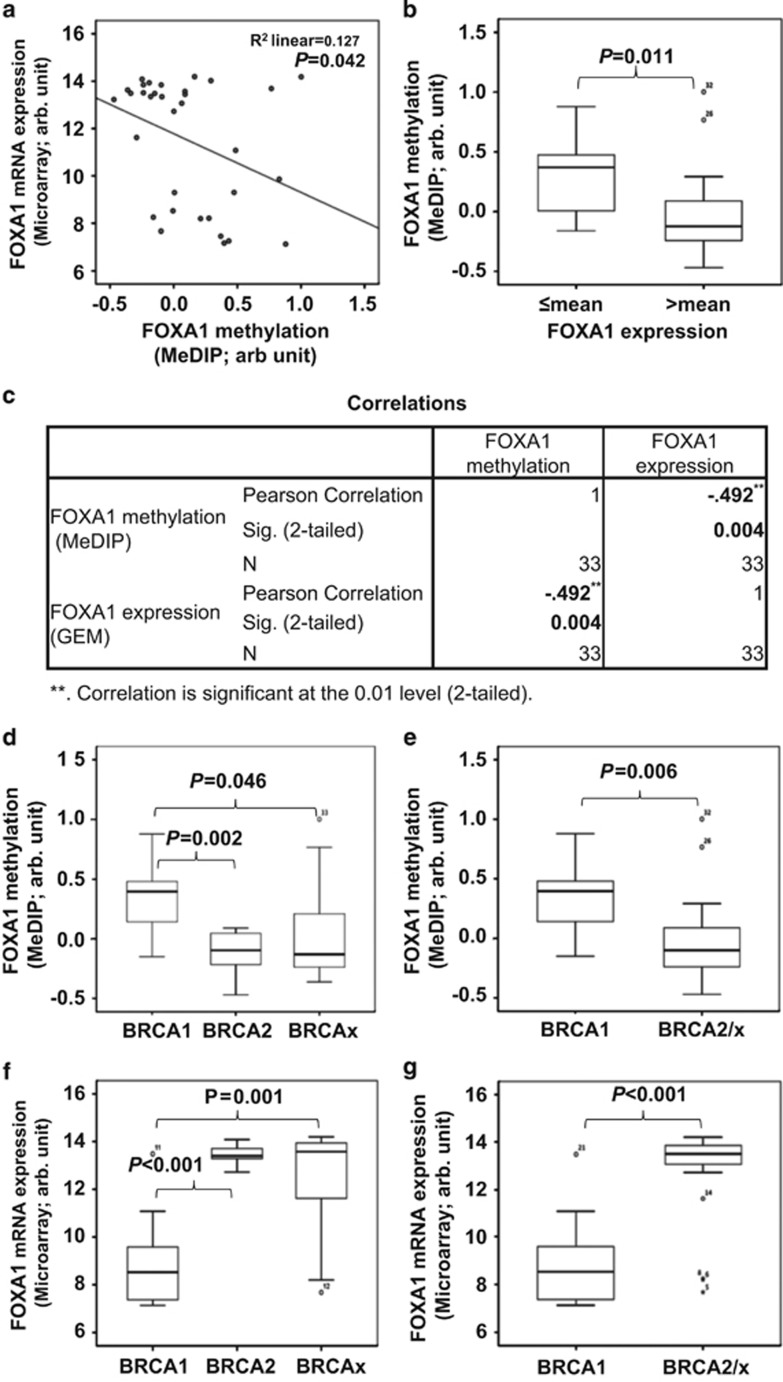
*FOXA1* gene promoter is hypermethylated in BRCA1 mutation tumours and its methylation is negatively correlated with its expression. Frequency of *FOXA1* methylation in clinical samples with mutations in BRCA1, BRCA2 and BRCAx tumours was analyzed using the kConFab database. (**a**) Significant higher percentage of FOXA1 promoter methylation in BRCA1-mutated tumours compared with BRCA2 or BRCAx mutated tumour. (**b** and **c**) FOXA1 expression level inversely correlates with FOXA1 methylation. Box plots represent median (centre line), interquartile range (box) and 95th percentiles (whisker), and samples outwith this range are represented as points. GEM, gene expression marker. (**d** and **e**) Significant higher percentage of FOXA1 promoter methylation in BRCA1-mutated tumours compared with BRCA2 or/and BRCAx mutated tumour. (**f** and **g**) Significant lower percentage of FOXA1 expression in BRCA1-mutated tumours compared with BRCA2 or/and BRCAx mutated tumour.

Having established that BRCA1 regulates FOXA1 methylation and silencing as well as the mechanism involved in human and MMECs, we next examined the association between FOXA1 methylation and expression *in vivo* in a published methylated DNA immunoprecipitation and gene expression microarray data set derived from familial breast tumour samples collected by kConFab (The Kathleen Cuningham Foundation Consortium for Research into Familial Breast Cancer, Melbourne, Australia).^23^ There are 33 patient samples in the cohort, 11 of which are with BRCA1 mutation. Statistical analysis of the expression and methylation profiles of *FOXA1* in these familial breast cancers revealed that there was a strong and significant inverse correlation between *FOXA1* methylation and expression using both linear regression model (*R*^2^=0.127, *P*=0.042; Figures 11a and b; Supplementary Figures S7 and S8) and Pearson's correlation model (cutoff is the mean, Pearson correlation coefficient=−0.492, *P*=0.004; Figure 11c), providing further physiological evidence that *FOXA1* methylation negatively regulates its expression in breast cancer patient samples. We also compared the levels of FOXA1 methylation in different familial breast tumours and found that FOXA1 methylation levels were significantly higher in BRCA1-mutated tumours compared with BRCA2, BRCAx (non-BRCA1/2) and BRCA2/x tumours (*P*=0.006, *P*=0.046 and *P*=0.006, respectively; Student's *t*-tests; Figures 10d and e, Supplementary Figures S9 and S10). Moreover, we also observed that FOXA1 expression was significantly lower in BRCA1-mutated tumours compared with BRCA2, BRCAx and BRCA2/x tumours (*P*<0.001, *P*=0.001 and *P*<0.001, respectively; Student's *t*-test; Figures 11f and g; Supplementary Figures S11 and S12). These *in vivo* data provide further proof that DNA methylation is a major mechanism for repressing FOXA1 expression in familial breast cancers and confirm a role for BRCA1 in regulating *FOXA1* methylation and expression.

## Discussion

Mutations to the breast cancer susceptibility gene *BRCA1* predispose women to a high lifetime risk of breast and ovarian cancer.^30^ BRCA1 is implicated in mammary epithelial cell differentiation and its deficiency associated with basal-like breast cancer subtype, yet the underlying mechanism involved is still not well understood. Moreover, although a tumour suppressive role of BRCA1 in DNA damage repair and cell cycle checkpoint has been demonstrated, the exact reason whereby BRCA1 deficiency causes predominantly cancers of the breast and ovary also remains largely unknown. BRCA1 mutation is associated with developing triple-negative breast cancer of the basal subtype. It is also a marker for breast cancer endocrine therapy-insensitive and poor prognosis. Conversely, FOXA1 is a pioneer transcription factor and a recognized marker for luminal subtype of breast cancer. It is also a predictor for hormonal responsiveness and good outcome in breast cancer.^10, 31^

In this study, we show that FOXA1 expression is negatively regulated by promoter methylation and silencing, and that BRCA1 regulates FOXA1 expression through suppressing its methylation in both human breast cancer cells and MMECs. FOXA1 expression is enhanced in basal but not in BRCA1-wild-type MCF-7 cells following treatment with 5'-aza-dC, supporting our hypothesis that methylation has a role in *FOXA1* silencing in the basal cell lines. Pyrosequencing further re-enforced that BRCA1-deficent mouse mammary cell lines are hypermethylated compared with those expressing wild-type BRCA1, despite the changes in levels of *FOXA1* methylation upon BRCA1 reconstitution are relatively small.

There is evidence to suggest that DNA methylation is gradually lost passively through many rounds of cell division^29^ and during breast epithelial cell immortalization.^32^ In agreement, bisulphite-converted PCR analysis showed that the *FOXA1* promoter region is hypomethylated in BRCA1-positive as well as negative human breast cancer cell lines (Supplementary Figure S4). Nevertheless, the breast cancer patient gene expression microarray and methylated DNA immunoprecipitation analyses clearly show that BRCA1 mutation is significantly associated with *FOXA1* gene promoter methylation and expression downregulation.

Interestingly, recent studies have demonstrated that DNA hypomethylation can cause the redistribution of the H3K27me3 epigenetic marks and derepression of PRC2-targeted genes.^33^ Conversely, DNA hypomethylation can also be coupled to repressive chromatin domain formation and gene silencing through the deposit of H3K27me3 in breast cancer.^29^ Our data suggest that *FOXA1* gene expression is negatively regulated by EZH2, a key subunit in PRC2, as chemical inhibition or siRNA-mediated depletion of EZH2 enhanced FOXA1 expression in Brca1-knockout MMECs and MCF-7 cells. EZH2 has been shown to epigenetically suppress target gene expression through modulating histone methylation, especially H3K27 trimetylation.^34, 35^ In agreement, our results show that the H3K27me3 repressive histone mark is highly enriched on the *FOXA1* promoter in the BRCA1-deficient MDA-MB-231, SUM131MO2 and MDA-MB-436 cells compared with the wild-type BRCA1-expressing MCF-7 cells. Importantly, the ChIP data also demonstrate that BRCA1 depletion enhances and EZH2 knockdown reduces H3K27me3 levels on the *FOXA1* promoter, further suggesting an antagonistic role of BRCA1 and EZH2 on *FOXA1* gene expression. However, in addition to regulating trimethylation of H3K27, an epigenetic mark for transcriptionally silent chromatin, EZH2 might also recruit DNMTs, including DNMT1/3a/3b, to enhance promoter methylation and gene silencing.^36, 37^ As BRCA1 has been shown to be able to bind EZH2 and negatively regulate PRC2 complex activity,^20^ we reason that BRCA1 can promote the transcription of *FOXA1* indirectly through binding to EZH2 subunit of the PRC2 complex and restraining its methyltransferase and gene silencing activity. In agreement, ChIP assays showed that BRCA1, EZH2, DNMT1/3a/3b and H3K27me3 are recruited to the promoter region of *FOXA1*. The low levels of recruitment of DNMT1/3a/3b probably reflect the fact that the *FOXA1* gene expression is predominantly repressed through H3K27me3-associated gene silencing mechanisms rather than DNA methylation. In addition, co-immunoprecipitation and ChIP–reChIP experiments also revealed that both BRCA1 and DNMT3b bind to EZH2 but do not exist in the same complexes, supporting the notion that BRCA1 binding inhibits the methyltransferase activity of EZH2/PRC2. Together these findings unveil two epigenetic mechanisms by which BRCA1 and EZH2 combined to regulate *FOXA1* expression in breast epithelial cells, namely through DNA methylation and H3K27me3-related heterochromatinization (Supplementary Figure S13). Consistent with the finding that EZH2 is involved in the epigenetic repression of *FOXA1*, a previous genome-wide siRNA screen also revealed *FOXA1* as one of 40 genes derepressed in human embryonic fibroblasts depleted of PRC2 components, including EZH2, EED and suppressor of zeste homologue (SUZ12).^38^ Subsequent ChIP analysis also showed that SUZ12 is specifically recruited to the *FOXA1* gene, which is enriched for H3K27me3.^38^ These findings are further endorsed by a number of studies, which also found that *FOXA1* expression is repressed by EZH2.^39, 40^

FOXA1, ERα and GATA3 are key transcription factors essential for normal mammary epithelial cell development and they function cooperatively to regulate the expression of genes essential for luminal mammary epithelial cell development.^13, 41, 42^ FOXA1, ERα and GATA3 co-express in normal breast luminal epithelial cells and their expression is controlled by complex cross-regulatory mechanisms. For example, GATA3 regulates the expression of both *ESR1* and *FOXA1* mRNA,^43, 44, 45^ ERα regulates *GATA3* mRNA expression,^44, 45^ whereas FOXA1 regulates *ESR1* but not *GATA3* expression.^9^ Moreover, recent genome-wide ChIP-sequencing studies also show that FOXA1 is involved in the regulation of ERα-mediated transcription by dictating the binding location and transcriptional activity of ERα.^9^ This interdependent relationship between FOXA1, ERα and GATA3 ensures that a loss of FOXA1 expression will disrupt the FOXA1–ERα–GATA3 transcriptional network and, therefore, luminal mammary epithelial cell development. Indeed, FOXA1 expression in breast cancers is predictive of luminal ERα-positive disease, and co-reexpression of ERα, FOXA1 and GATA3 has been shown to be able to reprogramme ER-negative breast cancer cell lines to regain hormonal sensitivity.^41^ In addition to promoting mammary luminal phenotype, FOXA1 might also have a more direct role in repressing the basal breast cancer phenotype. It has been shown that FOXA1 also inhibits the transcription of basal-type associated genes such as *CD58*, *ANXA1*, *JAG1* and *SOX9*, whereas the loss of FOXA1 leads to the derepression of these basal genes.^13^ These findings together highlight a critical role of FOXA1 in maintaining the luminal and repressing the basal phenotype. In light of these findings, collectively, our results lead us to propose a mechanistic model in which BRCA1 can promote FOXA1 transcriptional expression and thereby, breast cancer luminal subtype development through targeting EZH2 (Supplementary Figure S12). Conversely, when BRCA1 is silenced or mutated, the activity of EZH2 is restored, culminating in trimethylation of H3K27, recruitment of DNMTs to CpG islands, hypermethylation and silencing of *FOXA1* and ultimately, expression of basal genes and phenotype. In summary, our findings are of importance for understanding the aetiology of breast cancer subtypes, with potential implications for breast cancer diagnosis and therapy.

## Materials and methods

### Cell culture

The human breast carcinoma cell lines MCF-7, MDA-MB-231, MDA-MB-436, MDA-MB-468, HCC70 and SUM1315MO2 originated from the American Type Culture Collection (LGC standards, Middlesex, UK) and were authenticated by Cancer Research UK (London, UK). The Brca1 knockout (KB1P-3 12) and reconstituted (KB1P3R E3 and KB1P3R F4) murine breast epithelial cell lines have been described.^18^ Also see Supplementary Materials and methods.

### FuGENE6 transfection

Cells were seeded into six-well plates or 100-mm dishes to achieve ~60% confluency before transfection. Plasmid DNA was transfected using FuGENE 6 (Roche Diagnostics, West Sussex, UK) in a 3:1 ratio (μl of FuGENE: μg of DNA) following manufacturer's instructions.

### RNA interference with small interfering RNAs (siRNAs)

All siRNAs for the work were ON-TARGET*plus* SMARTpool siRNA purchased from Dharmacon Thermo Scientific (Lafayette, CO, USA). The SMARTpool siRNAs used in this study were: siBRCA1 (L-003461-00), siEZH2 (L-004218-00) and the ON-TARGET*plus* Non-Targeting Pool (D-001810-10). All siRNA pools were resuspended to 20 μM in 1x siRNA buffer before use.

### Statistical analyses

All statistical analyses, unless otherwise specified, were carried out using SPSS 16.0 (SPSS Inc., Chicago, IL, USA) and Windows XPs (Microsoft, Redmond, WA, USA). Where appropriate, a two-tailed independent sample *t*-test or a Pearson correlation analysis were performed.

### Pyrosequencing and PCR analysis of bisulphite-converted breast cancer cell line DNA

See Supplementary Materials and methods.

### ChIP, real-time quantitative PCR and co-immunoprecipitation

These procedures were performed as previously described.^46^ See also Supplementary Materials and methods.

### Western blot analysis

Cells were harvested for western blot analysis as described.^47^ See also Supplementary Materials and methods.

## Supplementary Material

Supplementary Figure Legends

Supplementary Figure1

Supplementary Figure2

Supplementary Figure3

Supplementary Figure4

Supplementary Figure5

Supplementary Figure6

Supplementary Figure7

Supplementary Figure8

Supplementary Figure9

Supplementary Figure10

Supplementary Figure11

Supplementary Figure12

Supplementary Figure13

## References

[bib1] 1Mullan PB, Quinn JE, Harkin DP. The role of BRCA1 in transcriptional regulation and cell cycle control. Oncogene 2006; 25: 5854–5863. 16998500 10.1038/sj.onc.1209872

[bib2] 2Buckley NE, Nic An tSaoir CB, Blayney JK, Oram LC, Crawford NT, D'Costa ZC et al. BRCA1 is a key regulator of breast differentiation through activation of Notch signalling with implications for anti-endocrine treatment of breast cancers. Nucleic Acids Res 2013; 41: 8601–8614. 23863842 10.1093/nar/gkt626 PMC3794588

[bib3] 3Liu S, Ginestier C, Charafe-Jauffret E, Foco H, Kleer CG, Merajver SD et al. BRCA1 regulates human mammary stem/progenitor cell fate. Proc Natl Acad Sci USA 2008; 105: 1680–1685. 18230721 10.1073/pnas.0711613105 PMC2234204

[bib4] 4Turner NC, Reis-Filho JS. Basal-like breast cancer and the BRCA1 phenotype. Oncogene 2006; 25: 5846–5853. 16998499 10.1038/sj.onc.1209876

[bib5] 5Hosey AM, Gorski JJ, Murray MM, Quinn JE, Chung WY, Stewart GE et al. Molecular basis for estrogen receptor alpha deficiency in BRCA1-linked breast cancer. J Natl Cancer Inst 2007; 99: 1683–1694. 18000219 10.1093/jnci/djm207 PMC6485437

[bib6] 6Tkocz D, Crawford NT, Buckley NE, Berry FB, Kennedy RD, Gorski JJ et al. BRCA1 and GATA3 corepress FOXC1 to inhibit the pathogenesis of basal-like breast cancers. Oncogene 2012; 31: 3667–3678. 22120723 10.1038/onc.2011.531

[bib7] 7Cirillo LA, Lin FR, Cuesta I, Friedman D, Jarnik M, Zaret KS. Opening of compacted chromatin by early developmental transcription factors HNF3 (FoxA) and GATA-4. Mol Cell 2002; 9: 279–289. 11864602 10.1016/s1097-2765(02)00459-8

[bib8] 8Sekiya T, Muthurajan UM, Luger K, Tulin AV, Zaret KS. Nucleosome-binding affinity as a primary determinant of the nuclear mobility of the pioneer transcription factor FoxA. Genes Dev 2009; 23: 804–809. 19339686 10.1101/gad.1775509 PMC2666343

[bib9] 9Carroll JS, Liu XS, Brodsky AS, Li W, Meyer CA, Szary AJ et al. Chromosome-wide mapping of estrogen receptor binding reveals long-range regulation requiring the forkhead protein FoxA1. Cell 2005; 122: 33–43. 16009131 10.1016/j.cell.2005.05.008

[bib10] 10Mehta RJ, Jain RK, Leung S, Choo J, Nielsen T, Huntsman D et al. FOXA1 is an independent prognostic marker for ER-positive breast cancer. Breast Cancer Res Treat 2012; 131: 881–890. 21503684 10.1007/s10549-011-1482-6

[bib11] 11Nakshatri H, Badve S. FOXA1 in breast cancer. Expert Rev Mol Med 2009; 11: e8. 19261198 10.1017/S1462399409001008

[bib12] 12Habashy HO, Powe DG, Rakha EA, Ball G, Paish C, Gee J et al. Forkhead-box A1 (FOXA1) expression in breast cancer and its prognostic significance. Eur J Cancer 2008; 44: 1541–1551. 18538561 10.1016/j.ejca.2008.04.020

[bib13] 13Bernardo GM, Bebek G, Ginther CL, Sizemore ST, Lozada KL, Miedler JD et al. FOXA1 represses the molecular phenotype of basal breast cancer cells. Oncogene 2013; 32: 554–563. 22391567 10.1038/onc.2012.62 PMC3371315

[bib14] 14Nakshatri H, Badve S. FOXA1 as a therapeutic target for breast cancer. Expert Opin Ther Targets 2007; 11: 507–514. 17373880 10.1517/14728222.11.4.507

[bib15] 15Chase A, Cross NC. Aberrations of EZH2 in cancer. Clin Cancer Res 2011; 17: 2613–2618. 21367748 10.1158/1078-0432.CCR-10-2156

[bib16] 16Vire E, Brenner C, Deplus R, Blanchon L, Fraga M, Didelot C et al. The Polycomb group protein EZH2 directly controls DNA methylation. Nature 2006; 439: 871–874. 16357870 10.1038/nature04431

[bib17] 17Denis H, Ndlovu MN, Fuks F. Regulation of mammalian DNA methyltransferases: a route to new mechanisms. EMBO Rep 2011; 12: 647–656. 21660058 10.1038/embor.2011.110 PMC3128952

[bib18] 18Puppe J, Drost R, Liu X, Joosse SA, Evers B, Cornelissen-Steijger P et al. BRCA1-deficient mammary tumor cells are dependent on EZH2 expression and sensitive to polycomb repressive complex 2-inhibitor 3-deazaneplanocin A. Breast cancer Res 2009; 11: R63. 19709408 10.1186/bcr2354 PMC2750125

[bib19] 19Gonzalez ME, DuPrie ML, Krueger H, Merajver SD, Ventura AC, Toy KA et al. Histone methyltransferase EZH2 induces Akt-dependent genomic instability and BRCA1 inhibition in breast cancer. Cancer Res 2011; 71: 2360–2370. 21406404 10.1158/0008-5472.CAN-10-1933 PMC3071296

[bib20] 20Wang L, Zeng X, Chen S, Ding L, Zhong J, Zhao JC et al. BRCA1 is a negative modulator of the PRC2 complex. EMBO J 2013; 32: 1584–1597. 23624935 10.1038/emboj.2013.95 PMC3671259

[bib21] 21Lehmann BD, Bauer JA, Chen X, Sanders ME, Chakravarthy AB, Shyr Y et al. Identification of human triple-negative breast cancer subtypes and preclinical models for selection of targeted therapies. J Clin Invest 2011; 121: 2750–2767. 21633166 10.1172/JCI45014 PMC3127435

[bib22] 22Ehrlich M, Lacey M. DNA methylation and differentiation: silencing, upregulation and modulation of gene expression. Epigenomics 2013; 5: 553–568. 24059801 10.2217/epi.13.43 PMC3864898

[bib23] 23Flanagan JM, Cocciardi S, Waddell N, Johnstone CN, Marsh A, Henderson S et al. DNA methylome of familial breast cancer identifies distinct profiles defined by mutation status. Am J Hum Genet 2010; 86: 420–433. 20206335 10.1016/j.ajhg.2010.02.008 PMC2833389

[bib24] 24Christman JK. 5-Azacytidine and 5-aza-2'-deoxycytidine as inhibitors of DNA methylation: mechanistic studies and their implications for cancer therapy. Oncogene 2002; 21: 5483–5495. 12154409 10.1038/sj.onc.1205699

[bib25] 25Wu L, Wang F, Xu R, Zhang S, Peng X, Feng Y et al. Promoter methylation of BRCA1 in the prognosis of breast cancer: a meta-analysis. Breast Cancer Res Treat 2013; 142: 619–627. 24258259 10.1007/s10549-013-2774-9

[bib26] 26Hsu NC, Huang YF, Yokoyama KK, Chu PY, Chen FM, Hou MF. Methylation of BRCA1 promoter region is associated with unfavorable prognosis in women with early-stage breast cancer. PLoS ONE 2013; 8: e56256. 23405268 10.1371/journal.pone.0056256 PMC3566056

[bib27] 27McCabe MT, Ott HM, Ganji G, Korenchuk S, Thompson C, Van Aller GS et al. EZH2 inhibition as a therapeutic strategy for lymphoma with EZH2-activating mutations. Nature 2012; 492: 108–112. 23051747 10.1038/nature11606

[bib28] 28Velichutina I, Shaknovich R, Geng H, Johnson NA, Gascoyne RD, Melnick AM et al. EZH2-mediated epigenetic silencing in germinal center B cells contributes to proliferation and lymphomagenesis. Blood 2010; 116: 5247–5255. 20736451 10.1182/blood-2010-04-280149 PMC3012542

[bib29] 29Hon GC, Hawkins RD, Caballero OL, Lo C, Lister R, Pelizzola M et al. Global DNA hypomethylation coupled to repressive chromatin domain formation and gene silencing in breast cancer. Genome Res 2012; 22: 246–258. 22156296 10.1101/gr.125872.111 PMC3266032

[bib30] 30Narod SA, Foulkes WD. BRCA1 and BRCA2: 1994 and beyond. Nat Rev Cancer 2004; 4: 665–676. 15343273 10.1038/nrc1431

[bib31] 31Albergaria A, Paredes J, Sousa B, Milanezi F, Carneiro V, Bastos J et al. Expression of FOXA1 and GATA-3 in breast cancer: the prognostic significance in hormone receptor-negative tumours. Breast Cancer Res 2009; 11: R40. 19549328 10.1186/bcr2327 PMC2716509

[bib32] 32Novak P, Jensen TJ, Garbe JC, Stampfer MR, Futscher BW. Stepwise DNA methylation changes are linked to escape from defined proliferation barriers and mammary epithelial cell immortalization. Cancer Res 2009; 69: 5251–5258. 19509227 10.1158/0008-5472.CAN-08-4977 PMC2697259

[bib33] 33Reddington JP, Perricone SM, Nestor CE, Reichmann J, Youngson NA, Suzuki M et al. Redistribution of H3K27me3 upon DNA hypomethylation results in de-repression of Polycomb target genes. Genome Biol 2013; 14: R25. 23531360 10.1186/gb-2013-14-3-r25 PMC4053768

[bib34] 34Li J, Hart RP, Mallimo EM, Swerdel MR, Kusnecov AW, Herrup K. EZH2-mediated H3K27 trimethylation mediates neurodegeneration in ataxia-telangiectasia. Nat Neurosci 2013; 16: 1745–1753. 24162653 10.1038/nn.3564 PMC3965909

[bib35] 35Bae WK, Hennighausen L. Canonical and non-canonical roles of the histone methyltransferase EZH2 in mammary development and cancer. Mol Cell Endocrinol 2014; 382: 593–597. 23684884 10.1016/j.mce.2013.05.002 PMC3843995

[bib36] 36Liu Z, Ren G, Shangguan C, Guo L, Dong Z, Li Y et al. ATRA inhibits the proliferation of DU145 prostate cancer cells through reducing the methylation level of HOXB13 gene. PLoS One 2012; 7: e40943. 22808286 10.1371/journal.pone.0040943 PMC3396626

[bib37] 37Martins-Taylor K, Schroeder DI, LaSalle JM, Lalande M, Xu RH. Role of DNMT3B in the regulation of early neural and neural crest specifiers. Epigenetics 2012; 7: 71–82. 22207353 10.4161/epi.7.1.18750 PMC3329505

[bib38] 38Bracken AP, Dietrich N, Pasini D, Hansen KH, Helin K. Genome-wide mapping of Polycomb target genes unravels their roles in cell fate transitions. Genes Dev 2006; 20: 1123–1136. 16618801 10.1101/gad.381706 PMC1472472

[bib39] 39Schuettengruber B, Chourrout D, Vervoort M, Leblanc B, Cavalli G. Genome regulation by polycomb and trithorax proteins. Cell 2007; 128: 735–745. 17320510 10.1016/j.cell.2007.02.009

[bib40] 40Lee TI, Jenner RG, Boyer LA, Guenther MG, Levine SS, Kumar RM et al. Control of developmental regulators by Polycomb in human embryonic stem cells. Cell 2006; 125: 301–313. 16630818 10.1016/j.cell.2006.02.043 PMC3773330

[bib41] 41Kong SL, Li G, Loh SL, Sung WK, Liu ET. Cellular reprogramming by the conjoint action of ERalpha, FOXA1, and GATA3 to a ligand-inducible growth state. Mol Syst Biol 2011; 7: 526. 21878914 10.1038/msb.2011.59 PMC3202798

[bib42] 42Bernardo GM, Lozada KL, Miedler JD, Harburg G, Hewitt SC, Mosley JD et al. FOXA1 is an essential determinant of ERalpha expression and mammary ductal morphogenesis. Development 2010; 137: 2045–2054. 20501593 10.1242/dev.043299 PMC2875844

[bib43] 43Kouros-Mehr H, Slorach EM, Sternlicht MD, Werb Z. GATA-3 maintains the differentiation of the luminal cell fate in the mammary gland. Cell 2006; 127: 1041–1055. 17129787 10.1016/j.cell.2006.09.048 PMC2646406

[bib44] 44Wilson BJ, Giguere V. Meta-analysis of human cancer microarrays reveals GATA3 is integral to the estrogen receptor alpha pathway. Mol Cancer 2008; 7: 49. 18533032 10.1186/1476-4598-7-49 PMC2430971

[bib45] 45Eeckhoute J, Keeton EK, Lupien M, Krum SA, Carroll JS, Brown M. Positive cross-regulatory loop ties GATA-3 to estrogen receptor alpha expression in breast cancer. Cancer Res 2007; 67: 6477–6483. 17616709 10.1158/0008-5472.CAN-07-0746

[bib46] 46Myatt SS, Kongsema M, Man CW, Kelly DJ, Gomes AR, Khongkow P et al. SUMOylation inhibits FOXM1 activity and delays mitotic transition. Oncogene 2014; 33: 4316–4329. 24362530 10.1038/onc.2013.546 PMC4096495

[bib47] 47Collado M, Medema RH, Garcia-Cao I, Dubuisson ML, Barradas M, Glassford J et al. Inhibition of the phosphoinositide 3-kinase pathway induces a senescence-like arrest mediated by p27Kip1. J Biol Chem 2000; 275: 21960–21968. 10791951 10.1074/jbc.M000759200

