# Supplementary material for: FOXA1 repression is associated with loss of BRCA1 and increased promoter methylation and chromatin silencing in breast cancer
Source: Oncogene. 2014 Dec 22;34(39):5012–24. doi: 10.1038/onc.2014.421 (PMC4430311; doi:10.1038/onc.2014.421)
Supplement: Supplementary Materials and Methods [file onc2014421x1.doc]

**Supplementary Materials and Methods**

**Cell culture**

The human breast carcinoma cell lines MCF-7, MDA-MB-231, MDA-MB-436, MDA-MB-468, HCC70 and SUM1315MO2 originated from the American Type Culture Collection (LGC standards, Middlesex, UK) and were authenticated by Cancer Research UK (London, UK). The Brca1 knockout (KB1P3 12) and reconstituted (KB1P3R E3 and KB1P3R F4) murine breast epithelial cell lines have been described ^18^. MCF-7 cells were maintained in Dulbecco’s modified eagle’s medium (DMEM) (Sigma-Aldrich, Poole, UK) supplemented with 10% foetal calf serum (FCS) (First Link Ltd, Birmingham, UK), 4 mM glutamine and 100 U/ml penicillin/streptomycin (Sigma-Aldrich, UK) unless indicated otherwise. The Brca1 knockout and reconstituted mouse cells were maintained in DMEM-F12 with GlutaMAX (Life Technologies, Paisley, UK) supplemented with 10% FCS, 50 U/ml penicillin, 50 μg/ml streptomycin, 5 μg/ml insulin, 5 ng/ml epidermal growth factor and 5 ng/ml cholera toxin from Vibrio cholera (all supplements from Sigma-Aldrich, UK). MDA-MB-436 and MDA-MB-468 cells were maintained in Leibovitz’s L-15 medium (Gibco, Life Technologies; 11415-049) supplemented with 10% horse serum (Invitrogen), HCC70 cells were cultured in RPMI 1640 (Gibco:21765-029) supplemented with 10% foetal calf serum (FCS), and SUM1315MO2 cells in Ham’s F12 Nutrient Mixture (Gibco: 21765-029) supplemented with 5% horse serum (Invitrogen), 10 mg/ml insulin (Sigma-Aldrich, UK) and 20ng/ml recombinant human EGF (Peprotech, London, UK).

**FuGENE6 transfection**

Cells were seeded into six well plates or 100 mm dishes to achieve approximately 60% confluency before transfection. Plasmid DNA was transfected using FuGENE 6 (Roche Diagnostics, West Sussex, UK) in a 3:1 ratio (μl of FuGENE: μg of DNA) following manufacturer’s instructions.

**RNA interference with small interfering RNAs (siRNAs)**

All siRNAs for the work were ON-TARGET*plus* SMARTpool siRNA purchased from Dharmacon Thermo Scientific (Lafayette, CO, USA). The SMARTpool siRNAs used in this study were: siBRCA1 (L-003461-00), siEZH2 (L-004218-00) and the ON-TARGET*plus* Non-Targeting Pool (D-001810-10). All siRNA pools were resuspended to 20 μM in 1x siRNA buffer prior to use.

**Pyrosequencing**

CpG islands are defined as regions of DNA greater than 200 bp with a GC content above 50% and a ratio of observed/expected CpG dinucleotides above 0.6. Using this model, genome-wide CpG islands have been predicted in humans and mice in silico and information is available on the UCSC Genome Bioinformatics website (http://genome.ucsc.edu/). The CpG islands on FOXA1 human and mouse promoters were identified on the UCSC browser and primers for PCR and pyrosequencing were designed with the pyroMark. Streptavidin sepharose HP beads (GE Healthcare, Buckinghamshire, UK) hold by vacuum on probes, were used to immobilise the biotinylated PCR amplicons through a series of steps whereby the vacuum probes were immersed into formulated solutions. The amplicons were denatured and washed using the PyroMark Q96 Vacuum Prep Workstation (Qiagen, UK), leaving only the desired single-stranded DNA attached to the beads. Subsequently, the template DNA was released into a designed 96 well plate holding the sequencing primers (15pmol/reaction). Pyrosequencing was performed in duplicate for each sample using the PyroGold Regent Kit (Qiagen, UK) on the PyroMark Q96 MD (Qiagen, UK) system according to the manufacturer’s instructions. The percentage of methylation of CpG sites for individual genes was calculated using the Pyro Q-CpG software (version 1.0.9) (Qiagen, UK). Only high-quality CpG sites, determined “passed” by the quality control thresholds of the Pyro Q-CpG software were considered. In addition, a commercially available fully methylated genomic DNA sample was used as a positive control (Zymo Research Corporation, Irvine, CA, USA) and in-house whole genome amplified genomic DNA (GE Healthcare, UK) used as an unmethylated negative control. The percentage of methylated DNA at the *FoxA1* promoter was calculated as the average of 2 CpG sites. Each sample had duplicates and the average of duplicates was also used in the analysis. For each region, one or two sequencing primers were subsequently designed and again CpGs within the primer sequence were avoided. Designed primers are as follows: for human *FOXA1* sense 5’-GTTTTGTAAATAAAGTGAGGGTT-3’, antisense 5’-biotin-CCTCCTCRTCCTCTCCCCATTTAT-3’ with sequencing primer 5’-AGGGTTTYGTTTGGGG-3’ or sense 5’-AGGAYGAGGAGGTGGGTATTT-3’, antisense 5’-biotin-CCCAATACAACCATCCAACCCTATA-3’ with sequencing primer 5’-GTAAGATTTATATTAGTTTAATTGT-3’ or 5’-GTTTYGTAGTTTTGGGTTTTT-3’ and for mouse *Foxa1*, sense 5’- GGGTGGGGGTTATATTGGAA-3’, antisense 5’-biotin-TAGTTTTTAATATTTTGGAGTTAGTTYGGT-3’ with sequencing primer 5’-GGGGGTTATATTGGAAG-3’ or sense 5’-GGTTTTTTAGYGGGTAAAGATAAGTGT-3’, antisense 5’-biotin-GGTAGGAGTTTATTTGTTTGAGTTT-3’ with sequencing primer 5’-GGGTAAAGATAAGTGTTTTTTT-3’ or 5’-TTAAAGTTTAGAGTTTTTTAAAAT-3’. Y stands for either C or T.

**PCR and analysis of Bisulfite Converted Breast Cancer Cell Line DNA**

500ng of DNA was bisulfite-converted (EZ DNA Methylation kit, Zymo Research) and then subjected to PCR amplification with an annealing temperature of 55oC (45-55 cycles) using primers specific for the TSS of FOXA1 (Forward: GAGGGGAGTTGAGTAGTTGTAG and Reverse: CACATCAACTCAACTACACTTACC) or a region approximately 2Kb upstream of the TSS (Forward: TTAGGTAGGTAGAAGTAGAGGGAG and Reverse: TCCTACTAAATAAAAAAAACCCTATC) . PCR reactions were checked on a gel and cleaned up using a PCR purification kit (QIAGEN) before being cloned into pGEM T-Easy (Promega) and sequenced from the SP6 primer. Sequencing was analyzed with the BiQ Analyzer (Bock et al 2005 Bioinformatics, PMID: 16141249).

**PCR and analysis of Bisulfite Converted Breast Cancer Cell Line DNA**

500ng of DNA was bisulfite-converted (EZ DNA Methylation kit, Zymo Research) and then subjected to PCR amplification with an annealing temperature of 55^o^C (45-55 cycles) using primers specific for the TSS of FOXA1 (Forward: GAGGGGAGTTGAGTAGTTGTAG and Reverse: CACATCAACTCAACTACACTTACC) or a region approximately 2Kb upstream of the TSS (Forward: TTAGGTAGGTAGAAGTAGAGGGAG and Reverse: TCCTACTAAATAAAAAAAACCCTATC). PCR reactions were checked on a gel and cleaned up using a PCR purification kit (QIAGEN) before being cloned into pGEM T-Easy (Promega) and sequenced from the SP6 primer. Sequencing was analyzed with the BiQ Analyzer (Bock et al 2005 Bioinformatics, PMID: 16141249).

**Co-immunoprecipitation**

For co-immunoprecipitation experiments cell lysates were prepared in IP buffer (1% Nonidet P-40, 150 mM NaCl, 50 mM Tris-HCl [pH 7.4], 10 mM NaF, 1 mM sodium orthovanadate, 10 mM N-ethyl-amide and protease inhibitors [Complete protease inhibitor cocktail; Roche, Lewes, UK]) and pre-cleared Dynabeads Protein A/G (A for rabbit antibodies, G for mouse antibodies, Invitrogen, Life Technologies, UK) were then washed in PBS three times prior to use. Cells were harvested, lysed in IP buffer, frozen in dry ice, and defrosted quickly by hand and centrifuged to remove cell debris. The supernatant was pre-cleaned with 20μl of beads for 4 hours and incubated with the specific primary antibody at 4^0^C for O/N with gentle rotating. On the second day, 30μl of beads were added to the mixture and incubated at 4^0^C for another 4 h. After incubation, the beads were washed five times with cold PBS and boiled at 100^0^C for 5 min to elute the proteins. Proteins were separated by SDS–PAGE gel electrophoresis, transferred to nitrocellulose membrane and hybridized with the antibodies same as Western blotting.

**Real-time quantitative PCR (RT-qPCR)**

Amount of transcripts were quantified by absolute quantification (AQ) Power SYBR Green PCR Master Mix (Applied Biosystems, Life Technologies, UK) and analysed by ABI7900 Sequence Detection System (Applied Biosystems). Housekeeping gene L19 was used as internal control for normalization.

**Sequences of primers used:**

| Gene | Forward | Reverse |
| --- | --- | --- |
| BRCA1 (human) | 5’-GCGGACTCCCAGCACAGA-3’ | 5’-GCTTCTTCCATTCTTTTCTCTCACACA -3’ |
| FOXA1 (human) | 5’-GCTGGACTTCAAGGCATACGA-3’ | 5’-GCTGGACTTCAAGGCATACGA-3’ |
| EZH2 (human) | 5’-ATGCGACTGAGACAGCTCAA -3’ | 5’-CGCTGTTTCCATTCTTGGTT-3’ |
| ERα (human) | 5’-TGATCAGGTCCACCTTCTAGAATG-3’ | 5’-CGCCAGACGAGACCAATCAT-3’ |
| L19 (human) | 5’-CGCCAGACGAGACCAATCAT-3’ | 5’-GCAGCCGGCGCAAA-3’ |
| Foxa1 (mouse) | 5’-GCAGCCGGCGCAAA-3’ | 5’-ACCTTGACGAAACAATCGTT-3’ |
| Ezh2 (mouse) | 5’-ACCTTGACGAAACAATCGTT-3’ | 5’-AGTCCCTGCTTCTCTGTCACTGT-3’ |
| L19 (mouse) | 5’-CCCGTCAGCAGATCAGGAA-3’ | 5’-GTCACAGGCTTGCGGATGA-3’ |

**Chromatin immunoprecipitation (ChIP)**

Forty μl of Dynabeads Protein A/G was washed with 200μl of TSE I buffer for three times and diluted with 40μl of TSE I buffer. 4μg of antibodies including anti-BRCA1 (Millipore, Merck, Feltham, UK; 07-434), EZH2 (Diagenode; C15410039), FOXA1 (Abcam; ab23738), Dnmt3b (Abcam; ab13604), H3K27me3 (Abcam; ab6002), and rabbit IgG control (DAKO, Ely, UK) were first separately diluted in Buffer D, mixed with diluted Dynabeads and then rotated O/N at 4^0^C Cells cultured in 10mm dish with 90% confluency were crosslinked with 1% formaldehyde for 10 min, rinsed with ice-cold PBS, incubated with 2.5M glycine for 5 min and harvested with 2ml of scrapping buffer. After a sequential wash with PBS, Buffer I and Buffer II, cell pellet was resuspended in 300 μl of Lysis buffer and sonicated in cold room. Supernatant was then diluted in 300 μl of Buffer D from which 100 μl was taken as INPUT. 200μl of cell lysate was mixed with prepared Dynabeads and rotated O/N at 4°C. After a sequential wash with TSE I, TSE II, Buffer III and TE buffer, 100 μl of elution buffer was added to the Dynabeads and the mixture was rotated at RT for 1 h. Eluted sample was collected in eppendorf and the Dynabeads was re-eluted with another 100 μl        crosslinked by incubating at 65°C O/N. PCR Purification Kit (Qiagen) was then used to purify DNA following manufacture’s instruction. Polymerase chain reaction (PCR) and Quantitative real-time PCR were performed with the following primers: FOXA1 FW:5’-AACAAGAAGATTTTGCGGATTC-3’ RV:5’-GACCGGGTGATAGGCAATTA-3’.

**Western blot analysis**

Protein concentration was determined by detergent-compatible (DC) protein assay (Bio-Rad). Twenty micrograms of protein were separated by sodium dodecyl sulphate-polyacrylamide gel electrophoresis (SDS-PAGE), transferred to nitrocellulose membrane and hybridized with the following antibodies: BRCA1 (Millipore; 07-434), EZH2 (Diagenode, Seraing, Belgium; C15410039), FOXA1 (Abcam, Cambridge, UK; ab23738), ERα (Santa-Cruz, Insight Biotechnology Ltd, Middlesex, UK; sc-7207), Dnmt1 (Abcam; ab13537), Dnmt3b (Abcam; ab13604), Dnmt3a (Abcam; ab13888) H3K27me3 (Abcam; ab6002).

**Statistical Analysis**

All statistical analyses, unless otherwise specified, were carried out using SPSS 16.0 (SPSS inc, Chicago, IL, USA) and Windows XP (Microsoft, Redmond, WA, USA). Where appropriate, a two-tailed independent sample t-test or a Pearson correlation analysis will be performed.
