# Supplementary material for: FOXA1 repression is associated with loss of BRCA1 and increased promoter methylation and chromatin silencing in breast cancer
Source: Oncogene. 2014 Dec 22;34(39):5012–24. doi: 10.1038/onc.2014.421 (PMC4430311; doi:10.1038/onc.2014.421)
Supplement: Supplementary Figure Legends [file onc2014421x2.doc]

**Supplement Figure Legends**

**Supplementary Figure S1**. BRCA1 correlates with FOXA1 expression in BRCA1 wild-type breast cancer cell panel. (a) Western blot and (b) qRT-PCR analysis was performed on a panel of 11 different BRCA1 wild-type human breast epithelial cell lines including MVF-10A, MCF-7, T47D, BT474.SKBR3, MDA-MB-231, HBL100, CAMA1, CAL51, ZR-7530 and ZR-75-1. The experiments were repeated three times independently and qRT-PCR results were normalized against L19 mRNA levels and the results presented as bars representing mean ± SD.

**Supplementary Figure S2**. KB1PR-3.12 E3 and KB1P-3.12 cells were treated with 0µM, 1µM and 5µM of 5’-aza-dC for 72 h with culture medium changed every day. Protein was extracted and expression of Ezh2 and Foxa1 were analyzed by western blotting. The experiments were repeated three times independently and gave similar results.

**Supplementary Figure S3**. Effects of EZH2 inhibition by GSK126 treatment on Foxa1 expression in murine BRCA1-deficient and BRCA1-reconstituted murine breast cancer cell lines. The BRCA1-defient murine breast cancer cell line KB1P-3.12 and the corresponding BRCA1-reconstituted murine cell line KB1PR-3.12 E3 were treated with 0µM, 1µM or 5µM of GSK126 for 72 h. Proteins were collected and expression of Ezh2 and Foxa1 were examined by western blot analysis. Foxa1 expression at the protein level did changed considerably in both cell lines after treatment.

**Supplementary Figure S4**. Methylation analysis of FOXA1 promoter in breast epithelial cell lines. a) Genomic context of FOXA1 showing analysed region and methylation levels in HMECs and HCC1964 cells. Shown is the region assessed for methylation by bsPCR. Methylation values are derived from whole genome bisulfite sequencing analysis of HMECs and HCC1954 cells (Hon *et al* 2012 Genome Research. 22:246-58, PMID: 22156296) The FOXA1 gene and CpG islands are also indicated below the plot. b) Region around FOXA1 TSS showing the location of the bsPRC amplicon used to for the FOXA1 TSS. c) Results of bsPCR analysis of FOXA1 TSS in breast cancer cell lines. Shown are individual clones (horizontal lines of beads) from bisulfite sequencing of DNA from different cancer cell lines. Methylated CpGs are indicated in black and unmethylated in white. Missing beads indicates missing data from the sequencing. The level of methylation observed is indicated below the cell line name. d) As for (c) except data from the upstream region is shown.

**Supplementary Figure S5.** EZH2 interacts with DNMT3a on the *FOXA1* promoter in MCF-7 and MDA-MB-236 cells. ChIP-reChIP assays were performed with BRCA1 and DNMT3a antibodies in MCF-7 and MDA-MB-236 cells. *; P ≤ 0.05 compared to IgG (mouse) controls.

**Supplementary Figure S6.** Empty expression vector control (pcDNA3) or the BRCA1 expression vector was transiently transfected into the basal-like MDA-MB-231 and MDA-MB-436 and the luminal MCF-7 cell lines. The transfected cells were then analyzed for FOXA1 expression by qRT-PCR **(a)** and Western blot analysis **(b).** The results show that BRCA1 reconstitution promotes FOXA1 mRNA expression in the basal-like MDA-MB-231 and MDA-MB-436 cell lines but not the luminal MCF-7 cell lines.

**Supplementary Figure S7. (a)** Scatter plot of FOXA1 methylation versus FOXA1 mRNA expression. BRCA1-mutated samples were labelled blue while other samples (BRCA2 and BRCAx) were labelled green. **(b)** Linear regression analysis for modeling the relationship between FOXA1 methylation and expression. There was significant negative linear relationship between FOXA1 methylation and FOXA1 mRNA expression level (p=0.042, R2= 0.127).

**Supplementary Figure S8. (a)** Means and medians of FOXA1 methylation and expression levels. The total number of cases is 33. **(b)** The 33 cases were categorized into two different groups according to the gene expression level of FOXA1 (GEM ≤mean and GEM >mean). The difference between means of FOXA1 methylation level of the two groups was statistically significant (students’ t-test, p=0.011).

**Supplementary Figure S9.** Comparisons of means of FOXA1 methylation levels between BRCA1 and BRCA2, and between BRCA1 and BRCAx. The mean FOXA1 methylation level of BRCA1 group was significantly higher when compared with BRCA2 or BRCAx (p=0.002 and p=0.046, respectively).

**Supplementary Figure S10.** Comparisons of means of FOXA1 methylation levels between BRCA1 and BRCA2/x groups. The mean of FOXA1 methylation in BRCA1 group was 0.363 while the mean of FOXA1 expression in BRCA2/x (others) group was -0.0148. The difference between the means were statistically significant by students’ t-test (p=0.006).

**Supplementary Figure S11.** Comparisons of means of FOXA1 expression between BRCA1 and BRCA2/x or between BRCA2 and BRCAx. The mean FOXA1 expression of BRCA1 group was significantly higher when compared with BRCA2 or BRCAx (p<0.001 and p=0.001, respectively).

**Supplementary Figure S12.** Comparison of means of FOXA1 expression between BRCA1 and BRCA2/x groups. The mean of FOXA1 expression in BRCA1 group was 8.98 while the mean of FOXA1 expression in BRCA2/x (others) group was 12.77. The difference between the means were statistically significant by students’ t-test (p<0.001).

**Supplementary Figure S13**. Proposed model of how BRCA1 silencing or mutation could affect FOXA1 expression and luminal to basal subtype transition. With the expression of wild-type BRCA1, the activity of EZH2 and PRC2 complex is inhibited, FOXA1 promoter is not hyper-methylated. In addition, wild-type BRCA1 may also activate transcription of FOXA1 gene. The combined outcome is the sustained FOXA1 expression and luminal phenotype. On the contrary, when BRCA1 is silenced or mutated, the inhibitory effect on EZH2 is released, leading to active tri-methylation of H3K27, recruitment of DNMT3b to CpG islands and hyper-methylation of FOXA1 promoter. As a result, FOXA1 expression is silenced and the cells develop a basal-like phenotype.
