# Supplementary material for: FOXA1 repression is associated with loss of BRCA1 and increased promoter methylation and chromatin silencing in breast cancer
Source: Oncogene. 2014 Dec 22;34(39):5012–24. doi: 10.1038/onc.2014.421 (PMC4430311; doi:10.1038/onc.2014.421)
Supplement: Supplementary Figure1 [file onc2014421x3.ppt]

## Slide 1
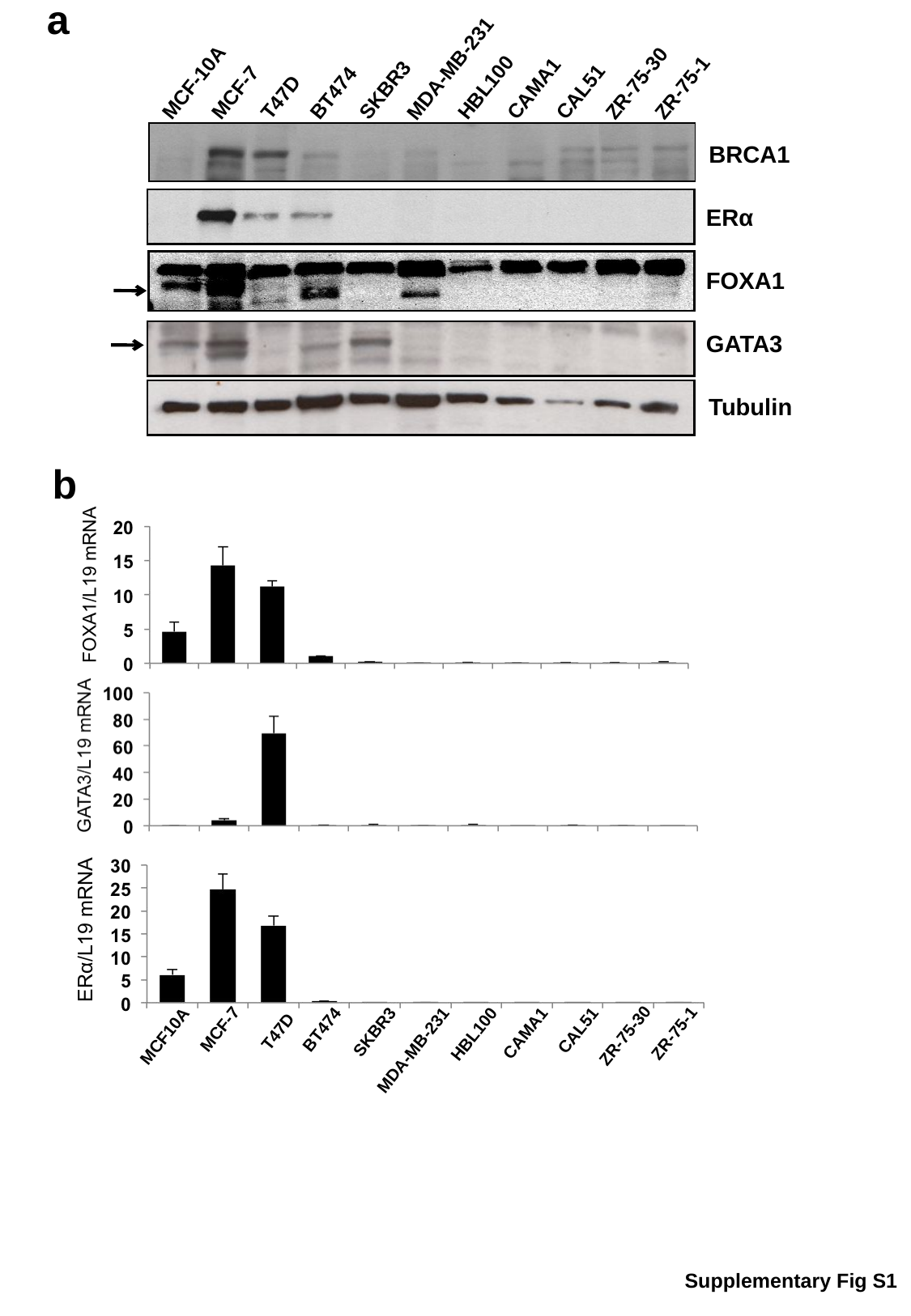

a
MDA-MB-231
MCF-10A
MCF-7
T47D
HBL100
CAMA1
CAL51
ZR-75-30
BT474
SKBR3
ZR-75-1
BRCA1
ERα
FOXA1
GATA3
Tubulin
b
ZR-75-30
ZR-75-1
MCF-7
MCF10A
BT474
SKBR3
HBL100
CAMA1
CAL51
T47D
MDA-MB-231
Supplementary Fig S1
