# Supplementary figures and images for: FOXA1 repression is associated with loss of BRCA1 and increased promoter methylation and chromatin silencing in breast cancer
Source: Oncogene. 2014 Dec 22;34(39):5012–24. doi: 10.1038/onc.2014.421 (PMC4430311; doi:10.1038/onc.2014.421)

## Slide 1
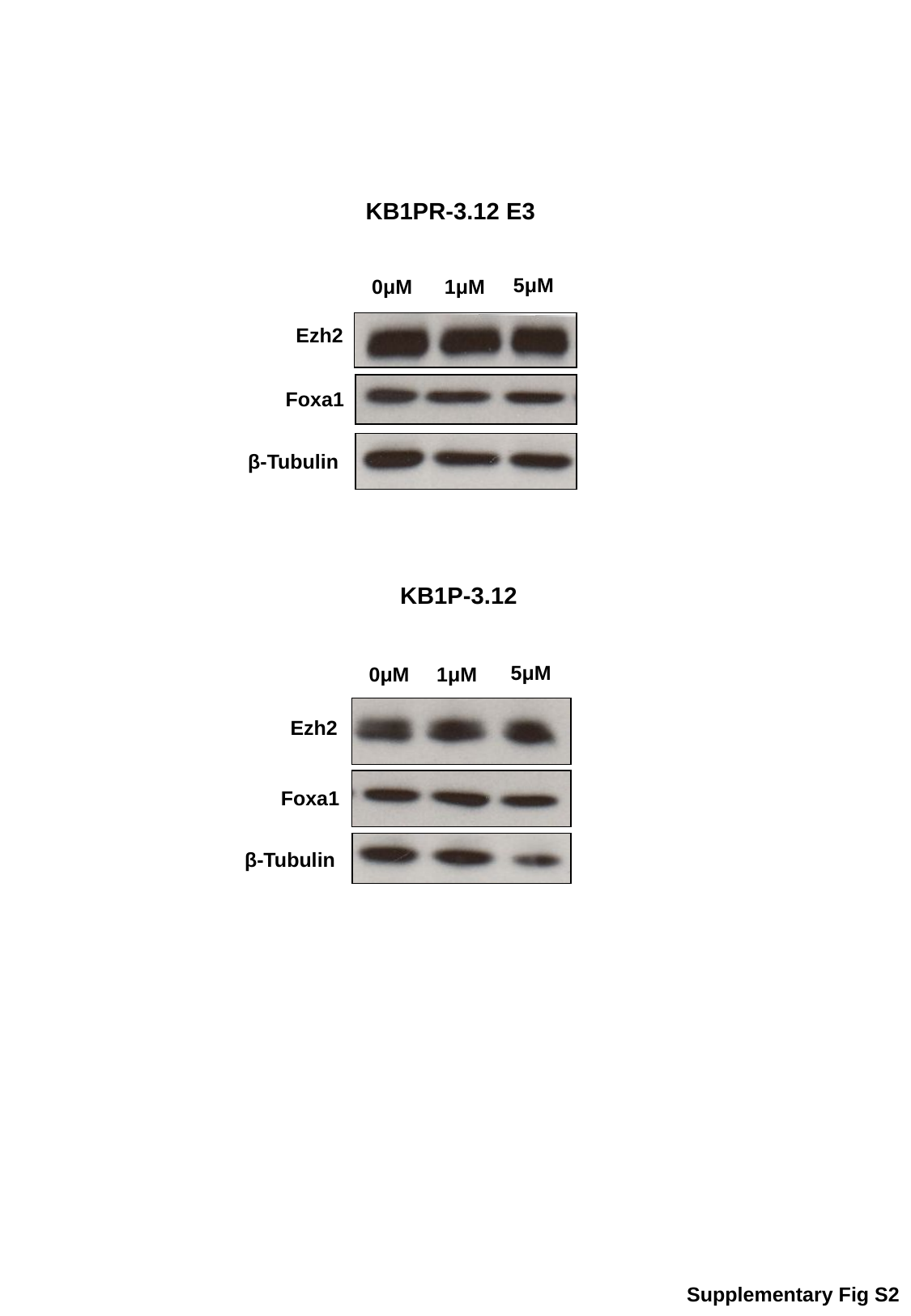

KB1PR-3.12 E3
5μM
0μM
Ezh2
Foxa1
β-Tubulin
1μM
KB1P-3.12
5μM
0μM
1μM
Ezh2
Foxa1
β-Tubulin
Supplementary Fig S2

Supplement: Supplementary Figure2 [file onc2014421x4.ppt]

## Slide 1
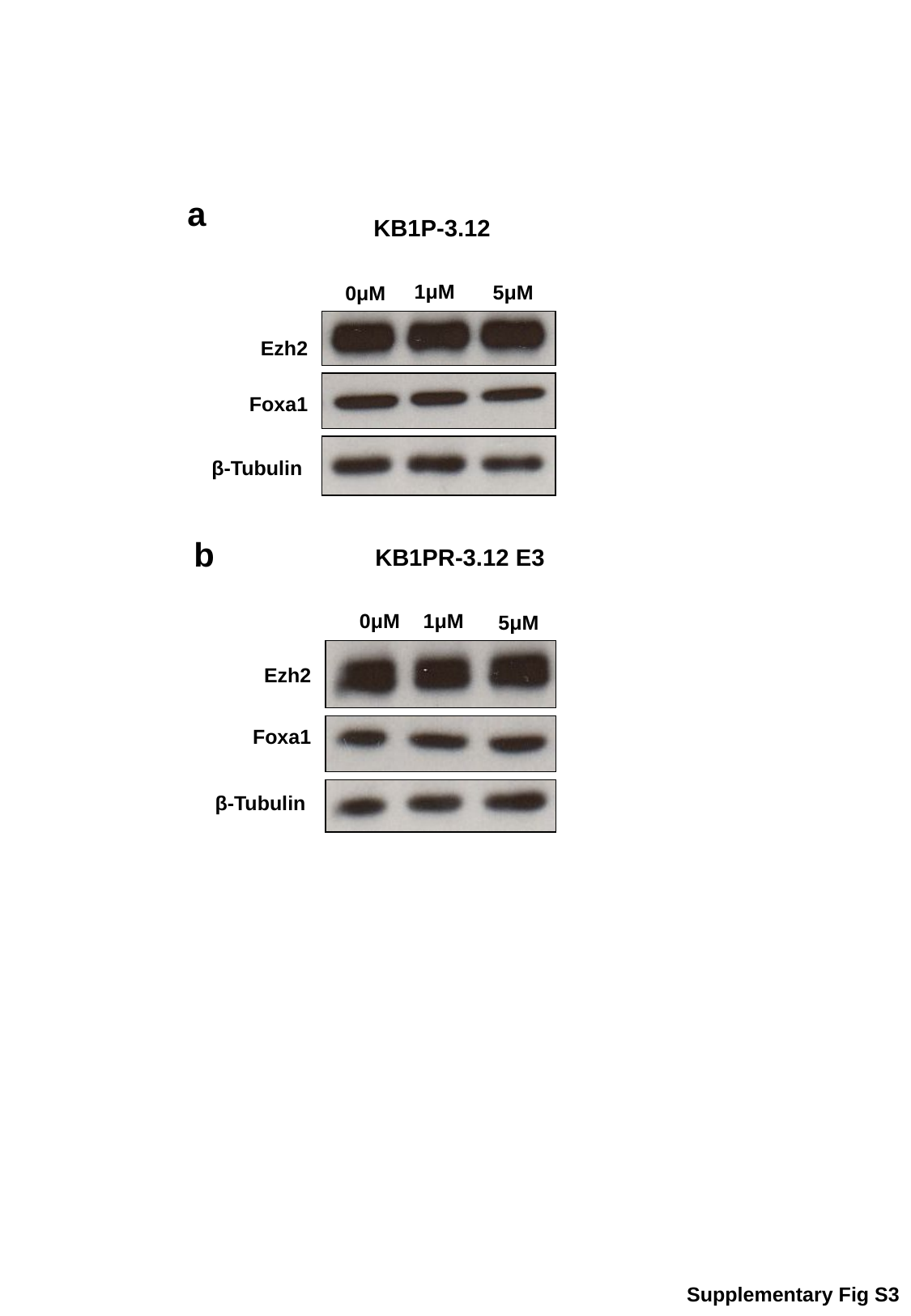

a
KB1P-3.12
1μM
5μM
0μM
Ezh2
Foxa1
β-Tubulin
b
KB1PR-3.12 E3
1μM
0μM
5μM
Ezh2
Foxa1
β-Tubulin
Supplementary Fig S3

Supplement: Supplementary Figure3 [file onc2014421x5.ppt]

## Slide 1
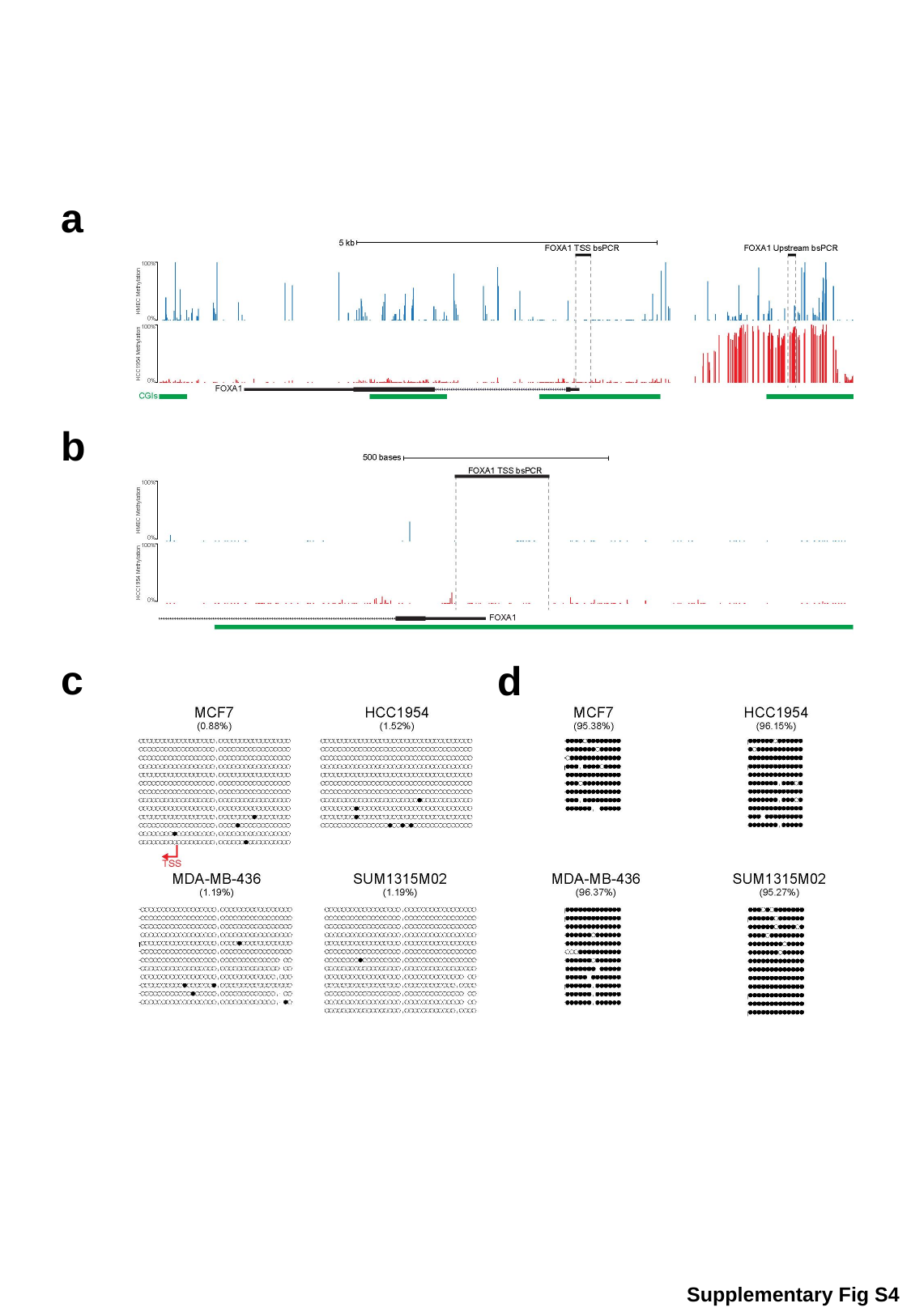

a
b
c
d
Supplementary Fig S4

Supplement: Supplementary Figure4 [file onc2014421x6.ppt]

## Slide 1
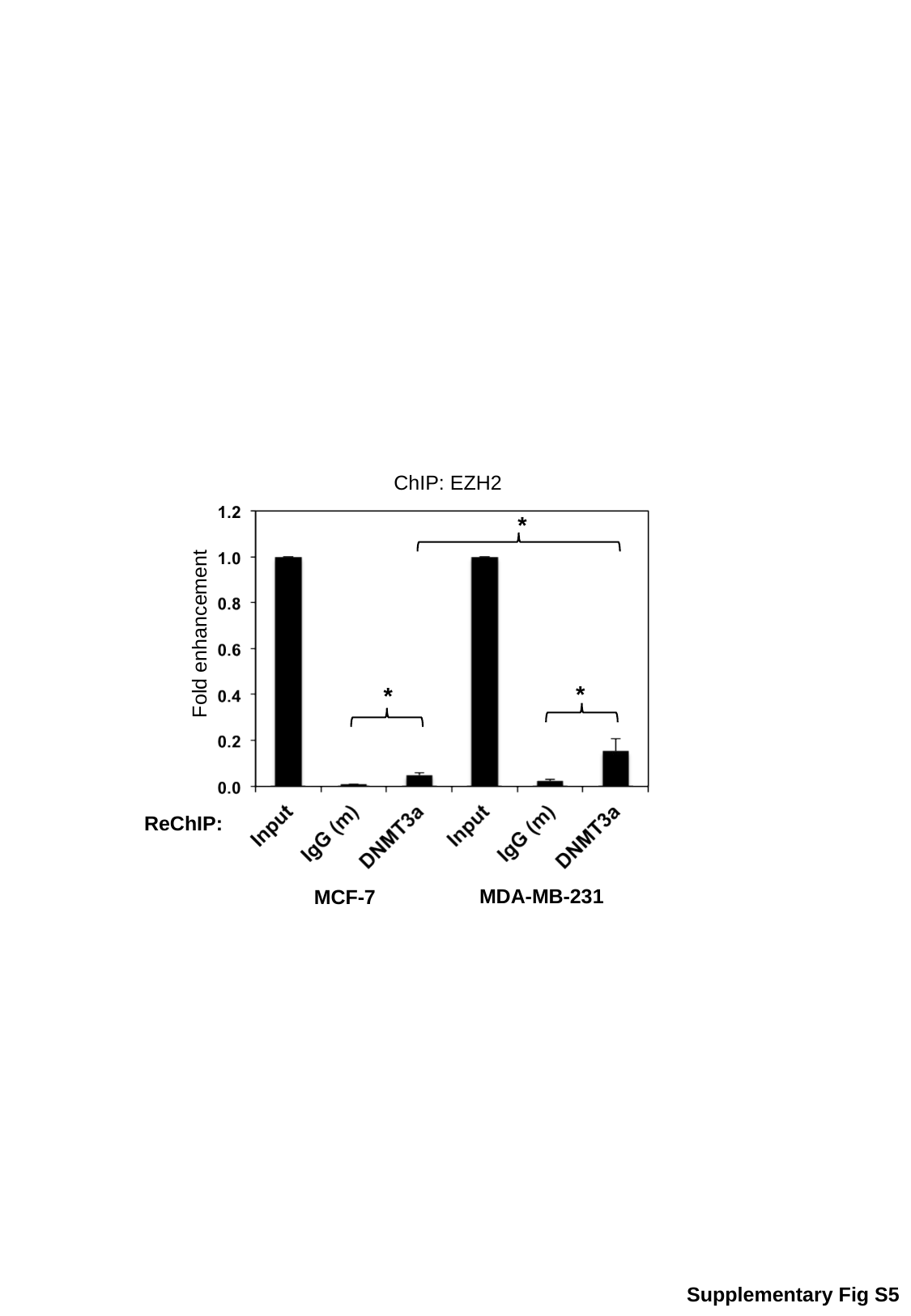

ChIP: EZH2
*
Fold enhancement
*
*
ReChIP:
MDA-MB-231
MCF-7
Supplementary Fig S5

Supplement: Supplementary Figure5 [file onc2014421x7.ppt]

## Slide 1
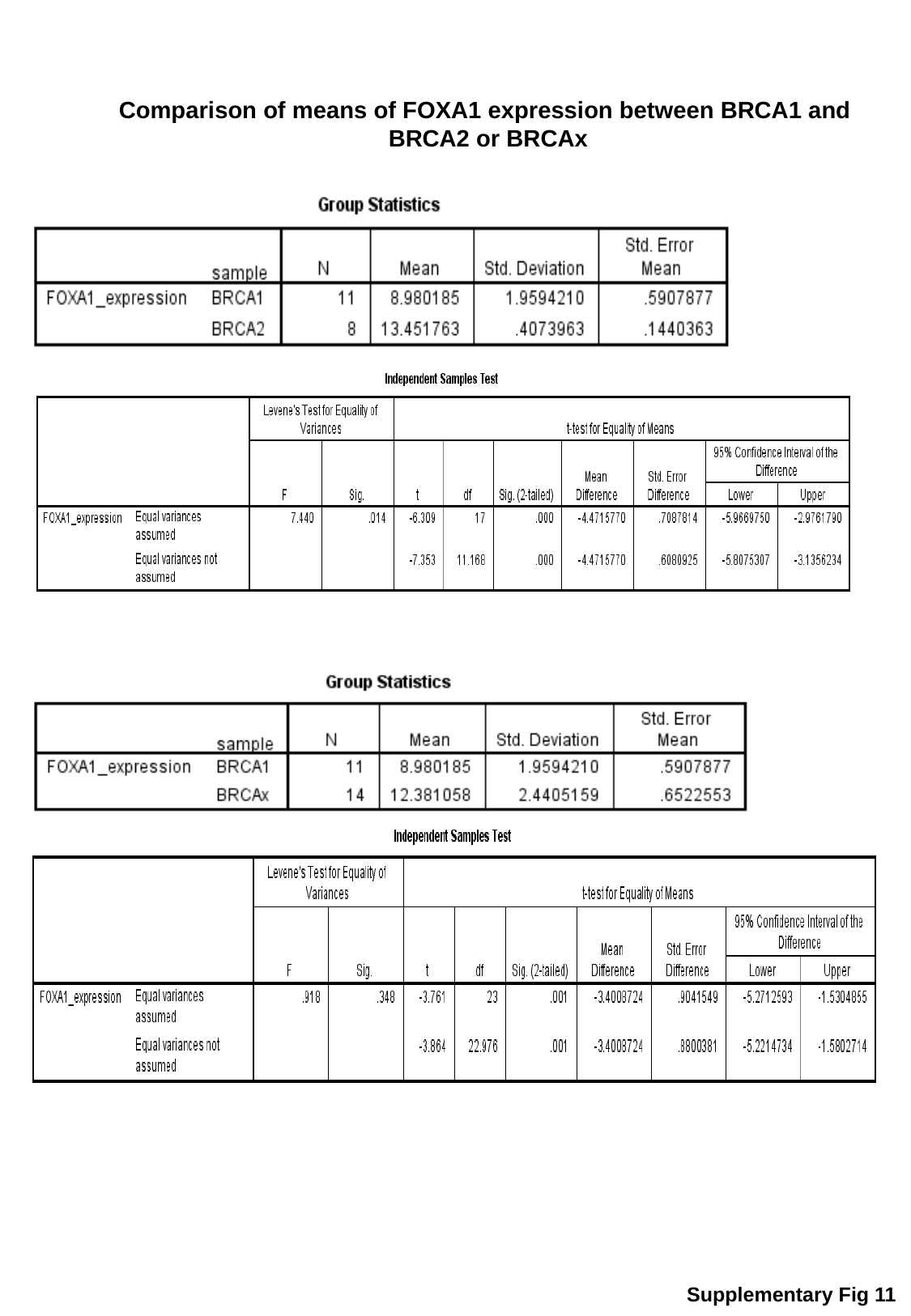

Comparison of means of FOXA1 expression between BRCA1 and
BRCA2 or BRCAx
Supplementary Fig 11

Supplement: Supplementary Figure11 [file onc2014421x13.ppt]
