# Supplementary material for: FOXA1 repression is associated with loss of BRCA1 and increased promoter methylation and chromatin silencing in breast cancer
Source: Oncogene. 2014 Dec 22;34(39):5012–24. doi: 10.1038/onc.2014.421 (PMC4430311; doi:10.1038/onc.2014.421)
Supplement: Supplementary Figure6 [file onc2014421x8.ppt]

## Slide 1
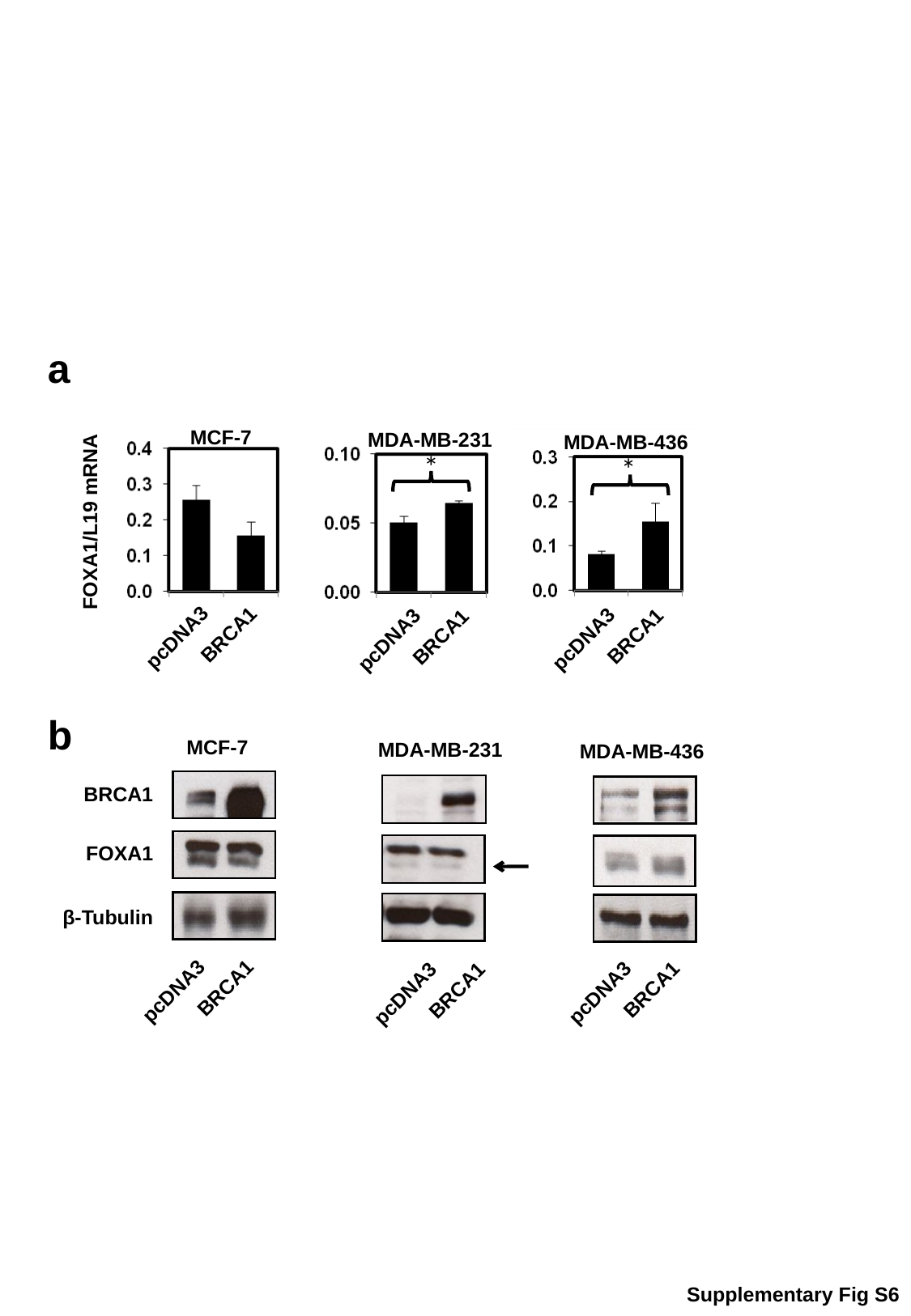

a
MCF-7
MDA-MB-231
MDA-MB-436
*
*
FOXA1/L19 mRNA
BRCA1
BRCA1
BRCA1
pcDNA3
pcDNA3
pcDNA3
b
MCF-7
MDA-MB-231
MDA-MB-436
BRCA1
FOXA1
β-Tubulin
BRCA1
BRCA1
BRCA1
pcDNA3
pcDNA3
pcDNA3
Supplementary Fig S6
