# Supplementary material for: FOXA1 repression is associated with loss of BRCA1 and increased promoter methylation and chromatin silencing in breast cancer
Source: Oncogene. 2014 Dec 22;34(39):5012–24. doi: 10.1038/onc.2014.421 (PMC4430311; doi:10.1038/onc.2014.421)
Supplement: Supplementary Figure7 [file onc2014421x9.ppt]

## Slide 1
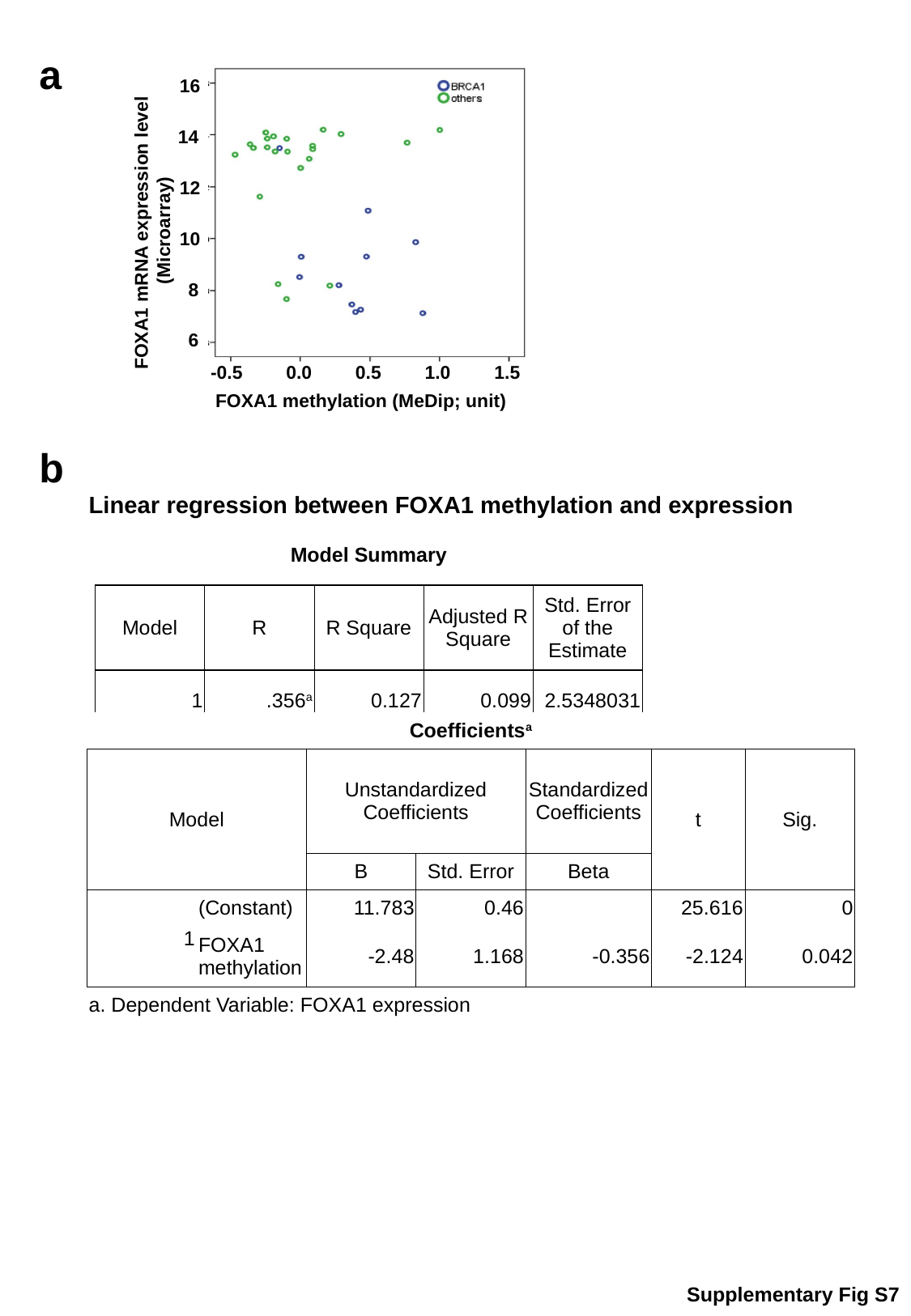

a
16
14
12
FOXA1 mRNA expression level
(Microarray)
10
8
6
-0.5
0.0
0.5
1.0
1.5
FOXA1 methylation (MeDip; unit)
b
| Linear regression between FOXA1 methylation and expression | | | | | | | |
| --- | --- | --- | --- | --- | --- | --- | --- |
| | Model Summary | | | | | | |
| | Model | R | R Square | Adjusted R Square | Std. Error of the Estimate | | |
| | 1 | .356a | 0.127 | 0.099 | 2.5348031 | | |
| | a. Predictors: (Constant), FOXA1 methylation | | | | | | |
| Coefficientsa | | | | | | |
| --- | --- | --- | --- | --- | --- | --- |
| Model | | Unstandardized Coefficients | | Standardized Coefficients | t | Sig. |
| | | B | Std. Error | Beta | | |
| 1 | (Constant) | 11.783 | 0.46 | | 25.616 | 0 |
| | FOXA1 methylation | -2.48 | 1.168 | -0.356 | -2.124 | 0.042 |
| a. Dependent Variable: FOXA1 expression | | | | | | |
Supplementary Fig S7
