# Supplementary material for: FOXA1 repression is associated with loss of BRCA1 and increased promoter methylation and chromatin silencing in breast cancer
Source: Oncogene. 2014 Dec 22;34(39):5012–24. doi: 10.1038/onc.2014.421 (PMC4430311; doi:10.1038/onc.2014.421)
Supplement: Supplementary Figure8 [file onc2014421x10.ppt]

## Slide 1
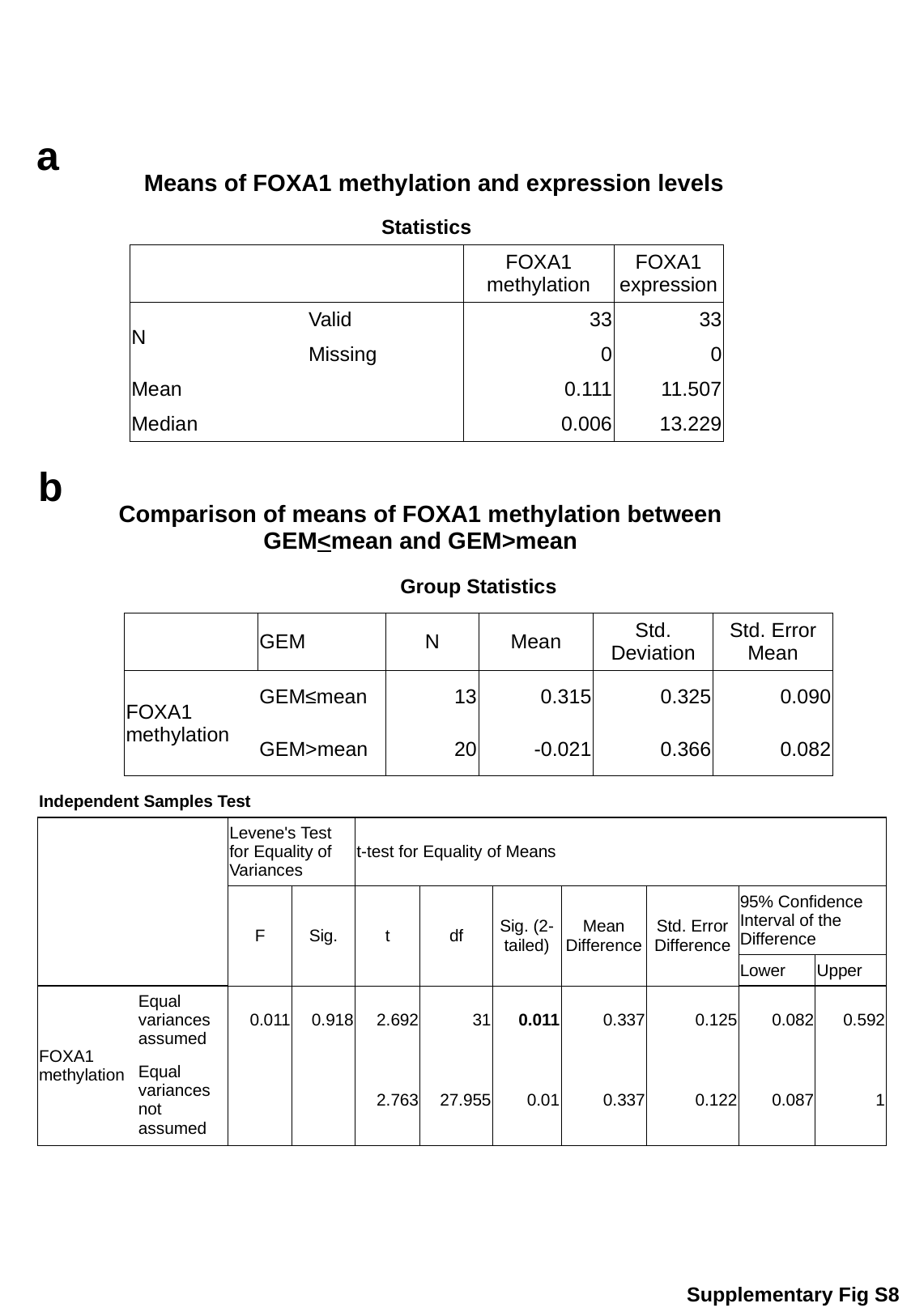

a
Means of FOXA1 methylation and expression levels
| Statistics | | | |
| --- | --- | --- | --- |
| | | FOXA1 methylation | FOXA1 expression |
| N | Valid | 33 | 33 |
| | Missing | 0 | 0 |
| Mean | | 0.111 | 11.507 |
| Median | | 0.006 | 13.229 |
b
| Comparison of means of FOXA1 methylation between GEM<mean and GEM>mean | | | | | | |
| --- | --- | --- | --- | --- | --- | --- |
| | Group Statistics | | | | | |
| | | GEM | N | Mean | Std. Deviation | Std. Error Mean |
| | FOXA1 methylation | GEM≤mean | 13 | 0.315 | 0.325 | 0.090 |
| | | GEM>mean | 20 | -0.021 | 0.366 | 0.082 |
| Independent Samples Test | | | | | | | | | | |
| --- | --- | --- | --- | --- | --- | --- | --- | --- | --- | --- |
| | | Levene's Test for Equality of Variances | | t-test for Equality of Means | | | | | | |
| | | F | Sig. | t | df | Sig. (2-tailed) | Mean Difference | Std. Error Difference | 95% Confidence Interval of the Difference | |
| | | | | | | | | | Lower | Upper |
| FOXA1 methylation | Equal variances assumed | 0.011 | 0.918 | 2.692 | 31 | 0.011 | 0.337 | 0.125 | 0.082 | 0.592 |
| | Equal variances not assumed | | | 2.763 | 27.955 | 0.01 | 0.337 | 0.122 | 0.087 | <number> |
Supplementary Fig S8
