# Supplementary material for: FOXA1 repression is associated with loss of BRCA1 and increased promoter methylation and chromatin silencing in breast cancer
Source: Oncogene. 2014 Dec 22;34(39):5012–24. doi: 10.1038/onc.2014.421 (PMC4430311; doi:10.1038/onc.2014.421)
Supplement: Supplementary Figure9 [file onc2014421x11.ppt]

## Slide 1
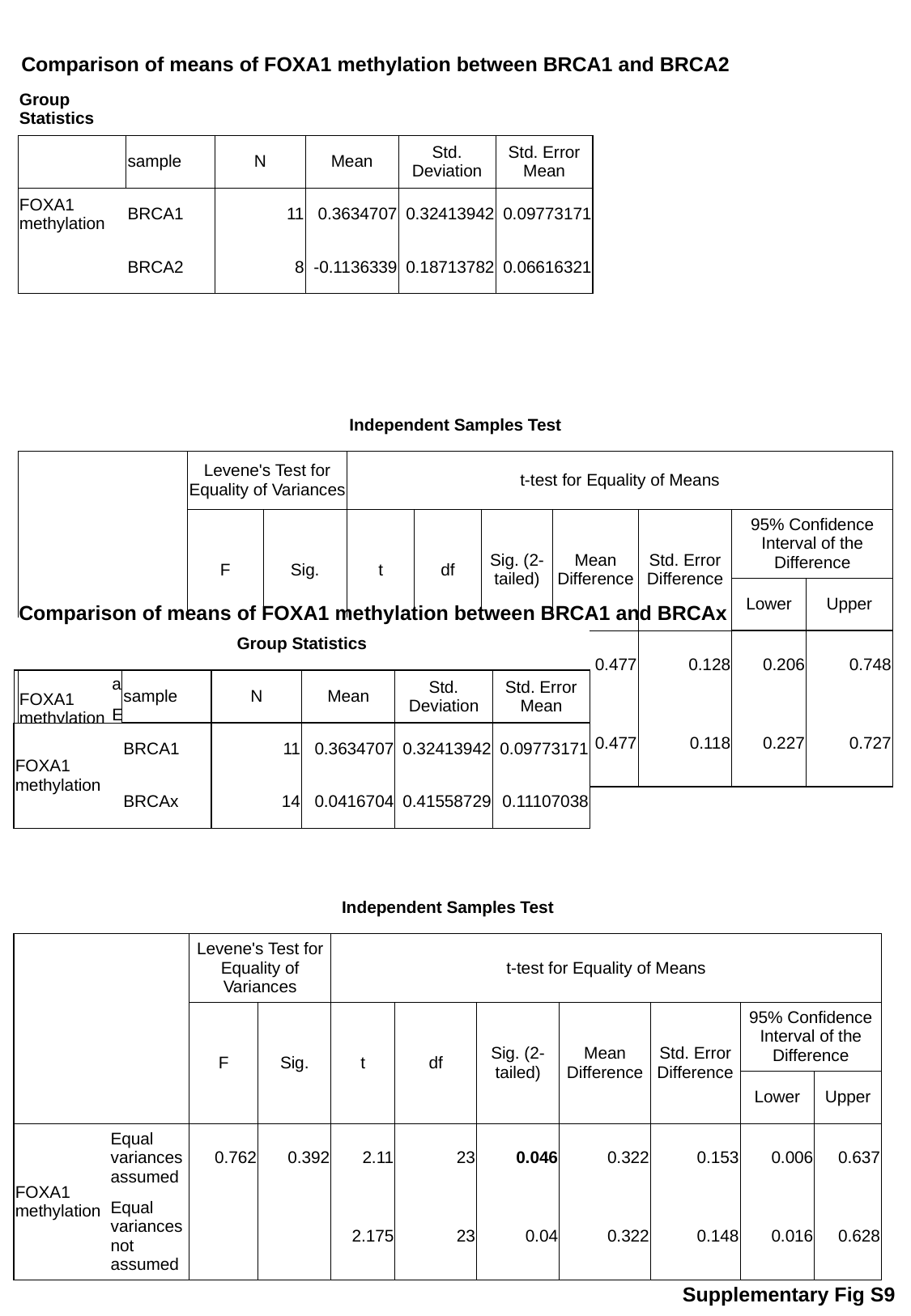

Comparison of means of FOXA1 methylation between BRCA1 and BRCA2
| | Group Statistics | | | | | | | | | | | | | | | | | | | | |
| --- | --- | --- | --- | --- | --- | --- | --- | --- | --- | --- | --- | --- | --- | --- | --- | --- | --- | --- | --- | --- | --- |
| | | | sample | | N | | Mean | | Std. Deviation | | | Std. Error Mean | | | | | | | | | |
| | FOXA1 methylation | | BRCA1 | | 11 | | 0.3634707 | | 0.32413942 | | | 0.09773171 | | | | | | | | | |
| | | | BRCA2 | | 8 | | -0.1136339 | | 0.18713782 | | | 0.06616321 | | | | | | | | | |
| | | | | | | | | | | | | | | | | | | | | | |
| | | | | | | | | | | | | | | | | | | | | | |
| | Independent Samples Test | | | | | | | | | | | | | | | | | | | | |
| | | | | Levene's Test for Equality of Variances | | | | t-test for Equality of Means | | | | | | | | | | | | | |
| | | | | F | | Sig. | | t | | df | Sig. (2-tailed) | | Mean Difference | | Std. Error Difference | | 95% Confidence Interval of the Difference | | | | |
| | | | | | | | | | | | | | | | | | Lower | | Upper | | |
| | FOXA1 methylation | Equal variances assumed | | 1.623 | | 0.22 | | 3.719 | | 17 | 0.002 | | 0.477 | | 0.128 | | 0.206 | | 0.748 | | |
| | | Equal variances not assumed | | | | | | 4.043 | | 16.358 | 0.001 | | 0.477 | | 0.118 | | 0.227 | | 0.727 | | |
Comparison of means of FOXA1 methylation between BRCA1 and BRCAx
| | Group Statistics | | | | | | | | | | | | | | | | | | | |
| --- | --- | --- | --- | --- | --- | --- | --- | --- | --- | --- | --- | --- | --- | --- | --- | --- | --- | --- | --- | --- |
| | | | sample | | N | | Mean | | Std. Deviation | | Std. Error Mean | | | | | | | | | |
| | FOXA1 methylation | | BRCA1 | | 11 | | 0.3634707 | | 0.32413942 | | 0.09773171 | | | | | | | | | |
| | | | BRCAx | | 14 | | 0.0416704 | | 0.41558729 | | 0.11107038 | | | | | | | | | |
| | | | | | | | | | | | | | | | | | | | | |
| | Independent Samples Test | | | | | | | | | | | | | | | | | | | |
| | | | | Levene's Test for Equality of Variances | | | | t-test for Equality of Means | | | | | | | | | | | | |
| | | | | F | | Sig. | | t | df | Sig. (2-tailed) | | Mean Difference | | Std. Error Difference | | 95% Confidence Interval of the Difference | | | | |
| | | | | | | | | | | | | | | | | Lower | | Upper | | |
| | FOXA1 methylation | Equal variances assumed | | 0.762 | | 0.392 | | 2.11 | 23 | 0.046 | | 0.322 | | 0.153 | | 0.006 | | 0.637 | | |
| | | Equal variances not assumed | | | | | | 2.175 | 23 | 0.04 | | 0.322 | | 0.148 | | 0.016 | | 0.628 | | |
| | | | | | | | | | | | | | | | | | | | | |
Supplementary Fig S9
