# Supplementary material for: FOXA1 repression is associated with loss of BRCA1 and increased promoter methylation and chromatin silencing in breast cancer
Source: Oncogene. 2014 Dec 22;34(39):5012–24. doi: 10.1038/onc.2014.421 (PMC4430311; doi:10.1038/onc.2014.421)
Supplement: Supplementary Figure10 [file onc2014421x12.ppt]

## Slide 1
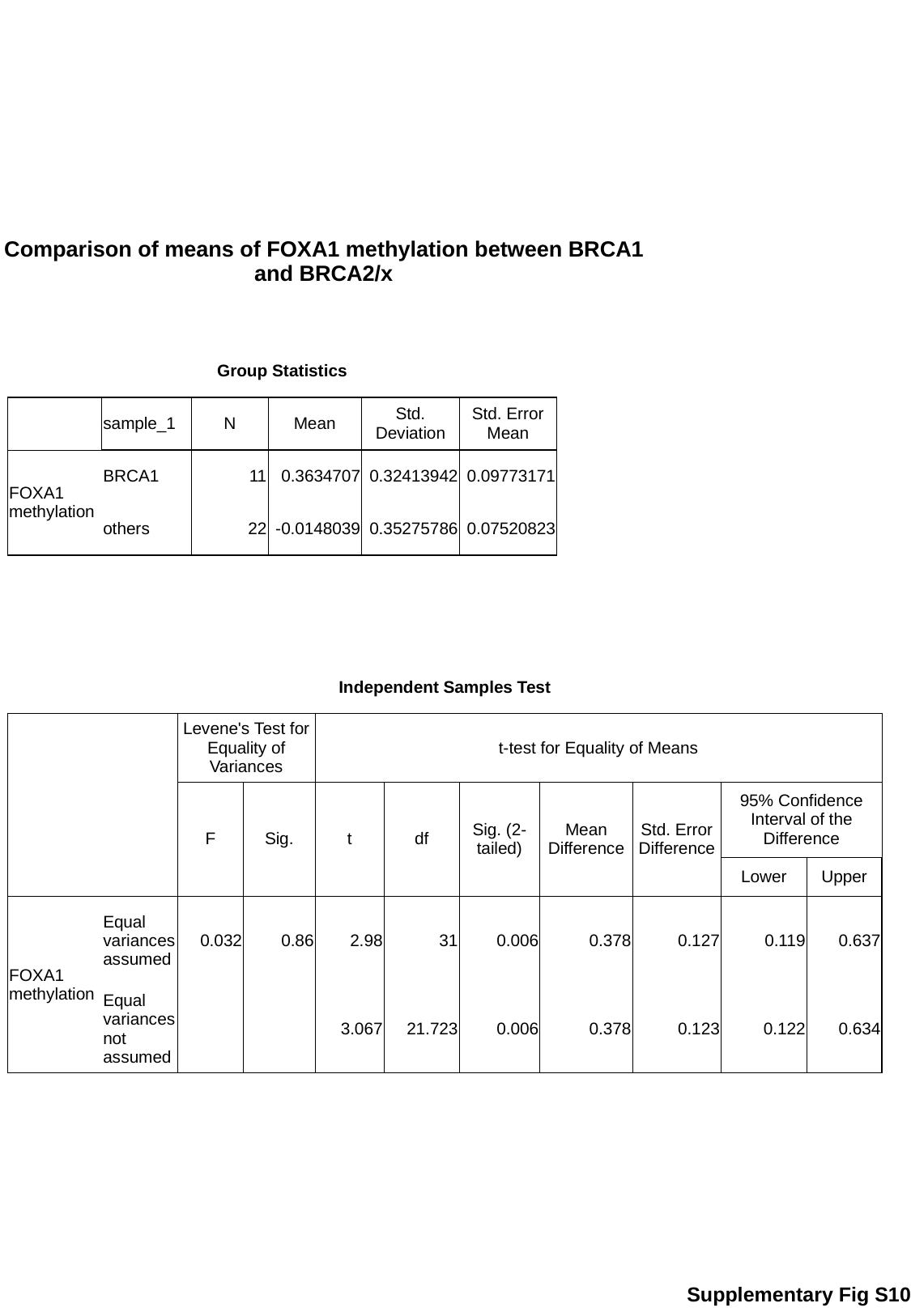

| Comparison of means of FOXA1 methylation between BRCA1 and BRCA2/x | | | | | | | | | | | | | | | | | | | | |
| --- | --- | --- | --- | --- | --- | --- | --- | --- | --- | --- | --- | --- | --- | --- | --- | --- | --- | --- | --- | --- |
| | | | | | | | | | | | | | | | | | | | | |
| | Group Statistics | | | | | | | | | | | | | | | | | | | |
| | | | sample\_1 | | N | | Mean | | Std. Deviation | | Std. Error Mean | | | | | | | | | |
| | FOXA1 methylation | | BRCA1 | | 11 | | 0.3634707 | | 0.32413942 | | 0.09773171 | | | | | | | | | |
| | | | others | | 22 | | -0.0148039 | | 0.35275786 | | 0.07520823 | | | | | | | | | |
| | | | | | | | | | | | | | | | | | | | | |
| | | | | | | | | | | | | | | | | | | | | |
| | Independent Samples Test | | | | | | | | | | | | | | | | | | | |
| | | | | Levene's Test for Equality of Variances | | | | t-test for Equality of Means | | | | | | | | | | | | |
| | | | | F | | Sig. | | t | | df | Sig. (2-tailed) | Mean Difference | | Std. Error Difference | | 95% Confidence Interval of the Difference | | | | |
| | | | | | | | | | | | | | | | | | | | | |
| | | | | | | | | | | | | | | | | Lower | | Upper | | |
| | FOXA1 methylation | | Equal variances assumed | 0.032 | | 0.86 | | 2.98 | | 31 | 0.006 | 0.378 | | 0.127 | | 0.119 | | 0.637 | | |
| | | | | | | | | | | | | | | | | | | | | |
| | | | Equal variances not assumed | | | | | 3.067 | | 21.723 | 0.006 | 0.378 | | 0.123 | | 0.122 | | 0.634 | | |
Supplementary Fig S10
