# Supplementary material for: FOXA1 repression is associated with loss of BRCA1 and increased promoter methylation and chromatin silencing in breast cancer
Source: Oncogene. 2014 Dec 22;34(39):5012–24. doi: 10.1038/onc.2014.421 (PMC4430311; doi:10.1038/onc.2014.421)
Supplement: Supplementary Figure12 [file onc2014421x14.ppt]

## Slide 1
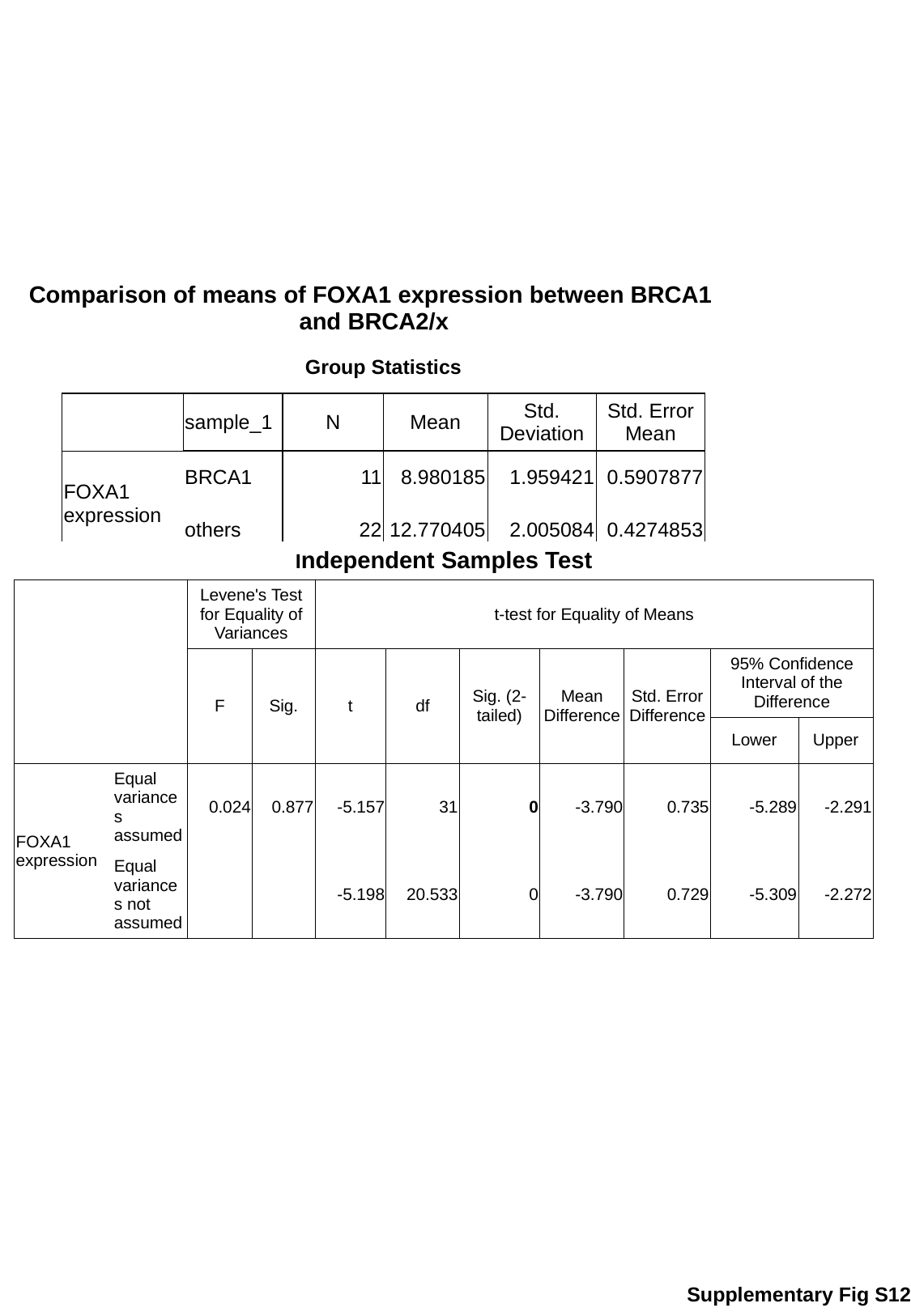

| Comparison of means of FOXA1 expression between BRCA1 and BRCA2/x | | | | | | | |
| --- | --- | --- | --- | --- | --- | --- | --- |
| | Group Statistics | | | | | | |
| | | sample\_1 | N | Mean | Std. Deviation | Std. Error Mean | |
| | FOXA1 expression | BRCA1 | 11 | 8.980185 | 1.959421 | 0.5907877 | |
| | | others | 22 | 12.770405 | 2.005084 | 0.4274853 | |
| Independent Samples Test | | | | | | | | | | |
| --- | --- | --- | --- | --- | --- | --- | --- | --- | --- | --- |
| | | Levene's Test for Equality of Variances | | t-test for Equality of Means | | | | | | |
| | | F | Sig. | t | df | Sig. (2-tailed) | Mean Difference | Std. Error Difference | 95% Confidence Interval of the Difference | |
| | | | | | | | | | Lower | Upper |
| FOXA1 expression | Equal variances assumed | 0.024 | 0.877 | -5.157 | 31 | 0 | -3.790 | 0.735 | -5.289 | -2.291 |
| | Equal variances not assumed | | | -5.198 | 20.533 | 0 | -3.790 | 0.729 | -5.309 | -2.272 |
| | | | | | | | | | | |
Supplementary Fig S12
