# Supplementary material for: FOXA1 repression is associated with loss of BRCA1 and increased promoter methylation and chromatin silencing in breast cancer
Source: Oncogene. 2014 Dec 22;34(39):5012–24. doi: 10.1038/onc.2014.421 (PMC4430311; doi:10.1038/onc.2014.421)
Supplement: Supplementary Figure13 [file onc2014421x15.ppt]

## Slide 1
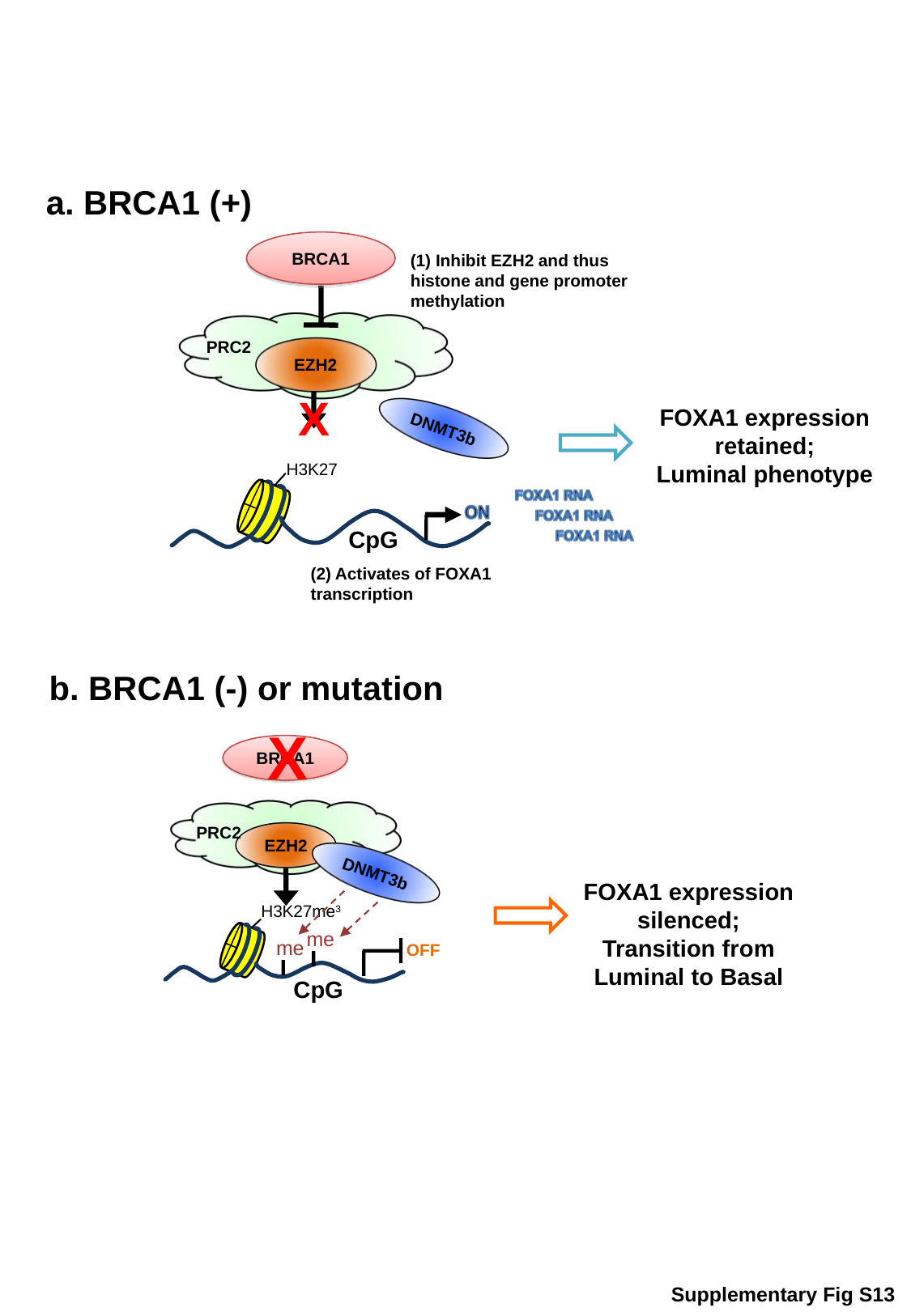

a. BRCA1 (+)
BRCA1
(1) Inhibit EZH2 and thus histone and gene promoter methylation
PRC2
EZH2
X
FOXA1 expression retained;
Luminal phenotype
DNMT3b
H3K27
CpG
(2) Activates of FOXA1 transcription
b. BRCA1 (-) or mutation
X
BRCA1
PRC2
EZH2
DNMT3b
FOXA1 expression silenced;
Transition from Luminal to Basal
H3K27me3
me
me
OFF
CpG
Supplementary Fig S13
